# Computational purification of individual tumor gene expression profiles leads to significant improvements in prognostic prediction

**DOI:** 10.1186/gm433

**Published:** 2013-03-28

**Authors:** Gerald Quon, Syed Haider, Amit G Deshwar, Ang Cui, Paul C Boutros, Quaid Morris

**Affiliations:** 1 Department of Computer Science, University of Toronto, 10 King's College Road, Room 3302, Toronto, ON, Canada, M5S 3G4; 2 Informatics and Biocomputing Platform, Ontario Institute for Cancer Research, 101 College Street, Suite 800, Toronto, ON, Canada, M5G 0A3; 3 Computer Laboratory, University of Cambridge, 15 JJ Thomson Avenue, Cambridge, United Kingdom, CB3 0FD; 4 Edward S. Rogers Sr. Department of Electrical and Computer Engineering, University of Toronto, 10 King's College Road, Room SFB540, Toronto, ON, Canada, M5S 3G4; 5 Division of Engineering Science, University of Toronto, 40 St. George Street, Suite 2110, Toronto, ON, Canada, M5S 2E4; 6 Department of Medical Biophysics, University of Toronto, 610 University Avenue, Room 7-411, Toronto, ON, Canada, M5G 2M9; 7 Department of Molecular Genetics, University of Toronto, 1 King's College Circle, Room 4396, Toronto, ON, Canada, M5S 1A8; 8 The Donnelly Centre, 160 College Street, Room 230, Toronto, ON, Canada, M5S 3E1; 9 Current address: Computer Science and Artificial Intelligence Laboratory, Massachusetts Institute of Technology, Cambridge, MA

## Abstract

Tumor heterogeneity is a limiting factor in cancer treatment and in the discovery of biomarkers to personalize it. We describe a computational purification tool, ISOpure, which directly addresses the effects of variable contamination by normal tissue in clinical tumor specimens. ISOpure uses a set of tumor expression profiles and a panel of healthy tissue expression profiles to generate a purified cancer profile for each tumor sample, and an estimate of the proportion of RNA originating from cancerous cells. Applying ISOpure before identifying gene signatures leads to significant improvements in the prediction of prognosis and other clinical variables in lung and prostate cancer.

## Background

Cancer patients with similar clinical and pathological characteristics can vary dramatically in their survival and response to treatment. Much of this variation is associated with differences in the molecular and cellular architecture of their tumors, suggesting that treatment decisions can be optimized based on molecular features of each individual's tumor [1]. Microarray and high-throughput sequencing technologies can profile the relative abundance of thousands of RNAs in a tumor, thereby providing a comprehensive snapshot of tumor state. These snapshots can increase the precision of patient categorizations that are traditionally based on type, size, spread, and histology [2]. Gene signatures derived from mRNA profiles have been used to identify cancer sub-types [3-5], to predict patient prognosis [6-11] and response to treatment [12,13], and to identify the site of origin [14,15]. Some of these signatures are already in routine clinical use [16-18] or undergoing trials [19].

Tumor samples drawn from patients usually exhibit significant cellular heterogeneity [2]. The proportion of healthy tissue in a sample can vary widely even among samples pre-selected to have a high cancerous cell content using pathological estimates [20-23], thereby introducing variability into expression profiles that cannot be removed by current computational pre-processing methods. This variability interferes with the development and clinical application of gene signatures by reducing the effective sample size of profiling studies, introducing confounding transcriptional signals even in moderately impure samples [24], and restricting the clinical use of gene signatures to tumor samples with sufficient cancerous cell content.

Post-operative methods for sample purification, such as laser capture micro-dissection or cell sorting, require specialized equipment, are costly, delay the diagnostic cycle, and cannot always be used. Furthermore, they may not remove all contaminating tissue, and can induce artificial cellular responses [25], while degrading samples and increasing the odds of sample confusion. A computational approach to purifying tumor profiles would address these issues.

It is possible to purify a single tumor profile computationally by representing it as a weighted average of its constituent (but hidden) cancer and non-cancerous 'normal' expression profiles, and then using statistical inference to jointly estimate both the mixture weights and the two constituent profiles. However, this is an under-determined system of equations, as there are more parameters than observations. Previous attempts to solve this problem can be viewed as different ways of regularizing these parameter estimates to make the problem well-determined. Most algorithms assume that multiple tumor samples in a collection are to be simultaneously purified, and that each of the tumor profiles in the collection is a mixture of a small number of shared cancer and normal profiles (that is, these algorithms constrain all normal and cancer expression profiles that constitute each tumor profile in the collection to be the same), and these methods represent each tumor profile only by their relative proportions of each shared profile [14,20,21,26-32]. Another approach assumes that the profile, ***h***_*n*_, of the contaminating normal cells can be measured separately for all cancer patients (whose tumor profiles are indexed by *n*) [33,34], and thus fixes ***h***_*n *_to the observed value, and freely estimates the cancer profile ***c***_*n *_of each tumor profile *n *as well as the mixing proportions. The former group of methods is not amenable to downstream sample-specific analyses such as prognostic prediction because they apply too strong regularization, and estimate only a handful of patient-specific covariates. The latter group of methods requires an accurate, separate measurement of ***h***_*n*_; these measurements are rarely available in archival datasets and are not always feasible to obtain in a clinical setting. Furthermore, the sensitivity of these methods to biological variability or measurement noise in the provided profile for ***h***_*n *_remains unclear and has never been tested. However, it seems likely that the sensitivity would be high, because the estimates of the cancer profiles ***c***_*n *_are not regularized and therefore incorporate any noise or error in the provided normal profile ***h***_*n*_.

In this paper, we describe a new approach to computational purification, called ISOpure, which, unlike previous approaches, is able to estimate a distinct cancer profile for each tumor sample; this cancer profile is robust to noise and does not require a matching normal profile. Using a dataset of 834 lung and prostate tumors, we found that ISOpure reduces inter-tumor variability caused by non-cancerous tissue contamination, leading to a significant increase in the power and accuracy of clinical prediction models. Using ISOpure to preprocess non-small cell lung adenocarcinoma expression profiles, we produced a validated gene signature that is a statistically significant predictor of prognosis for all lung adenocarcinoma tumors and for stage I tumors only.

## Methods

### The challenge of computational purification

The challenge of computational purification is to decompose each tumor profile ***t***_*n *_(a vector of length *G*) into its component cancer profile (the vector ***c***_*n*_), and normal profile (the vector ***h***_*n*_), and estimate a scalar, *α_n_*, that represents the fraction of the tumor sample RNA that was contributed by cancer cells. This estimation is typically made using a procedure that sets the parameters (***c***_*n*_, ***h***_*n*_, and *α_n_*) in order to minimize the reconstruction error, represented here by the vector ***e***_*n*_:

(1) $$t_{n}=\alpha_{n}c_{n}+\left(1-\alpha_{n}\right)h_{n}+e_{n}$$

Without further constraints on the parameters, equation (1) is an ill-defined problem because there are *2G+1 *parameters to estimate (***c***_*n*_, ***h***_*n*_, and *α_n_*) but only *G *observations (***t***_*n*_) so there is a continuum of solutions that satisfy equation (1) with zero error, suggesting that these solutions are over-fitting the problem. Computational purification methods apply different 'hard' and 'soft' constraints (also known as regularizations) to the parameters to ensure a unique, interpretable solution. Regularization strategies score the parameters based on how well they reflect prior assumptions about their likely values. For example, ISOpure assumes that the vector ***h***_*n *_is similar to one or more profiles of normal tissue that are input into the algorithm. Because the choice of regularization determines the solution to equation (1), the success of a computational purification method depends on the suitability of the regularizations that it applies. In the Results section, we evaluate ISOpure and other regularization strategies based on how much they improve prognostic models applied to the tumor profiles and how well they reproduce pathological evaluations of the tumors. Typically, good regularization strategies introduce a sufficiently strong bias into parameter estimation that they favor solutions to equation (1) that have non-zero error and therefore avoid over-fitting. Thus, assumptions about the distribution of ***e***_*n *_also influence the solution to equation (1). Different assumptions lead to different objective functions in the estimation, and can lead to different optimization procedures.

The following sections describe our ISOpure method in detail. In brief, ISOpure is based on a statistical model that represents the tumor profile as a sample from a multinomial distribution. The multinomial distribution is parameterized by a discrete probability distribution (represented by the vector ${\hat{x}}_{n}$) that ISOpure attempts to decompose into the cancer profile ***c***_*n *_and the normal profile ***h***_*n*_. The reconstruction error ***e***_*n *_from equation (1) can be interpreted as sampling noise from the multinomial distribution, but it is not explicitly represented in the ISOpure model. ISOpure makes two prior assumptions to avoid over-fitting: it assumes that ***h***_*n *_is a convex combination of the normal profiles provided to the algorithm, and that the cancer profiles ***c***_1_, ***c***_2_,..., ***c***_*N *_in the cohort are clustered together around a 'reference cancer profile', ***m***, which is also inferred from the data. The parameters of the ISOpure model, which include the individual cancer and normal profiles and the reference cancer profile, are fit by maximum *a posteriori *(MAP) estimation in the statistical model (equations 3 to 9) that encodes the ISOpure assumptions.

### ISOpure overview

Below we provide a brief overview of the major features of the algorithm. The following sections contain a description of the parameters of the ISOpure model, a formal specification of this statistical model (equations 3 to 9) and a step-by-step guide to the inference procedure that ISOpure uses to fit its model. Our notation is as follows: lower case letters (for example, *α_n_*) represent scalar parameters or indices, bold lowercase letters (for example, ***c***_*n*_) represent column vectors (which could be inputs or parameters), capital letters (for example, *G*) represent scalar constants, and bold capital letters (for example, ***B***) represent matrices.

#### ISOpure inputs

In our comparisons, the following input data were assumed to be available to ISOpure and other algorithms:

a) ***t***_1_, ***t***_2_,..., ***t***_*N *_is a set of *N *tumor profiles. Each profile is represented by a vector of *G *non-negative (that is, 0 or greater) elements, where each element represents the measured expression level of a transcript. *G *is typically on the order of 10,000. Microarray intensities should be normalized but not log-transformed before input into ISOpure, as the algorithm interprets each element as a normalized count of the number of copies of each transcript present in the sample.

b) ***b***_1_, ***b***_2_,..., ***b***_*R *_is a set of *R *healthy profiles, defined as above, that are ideally collected using the same protocol as was used to collect the tumor profiles. Note that we expect *R *to be less than *N*, and we do not require that any of the healthy profiles be matched to a tumor.

#### ISOpure outputs

ISOpure estimates the following variables from the input data:

a) ***c***_1_, ***c***_2_,..., ***c***_*N *_is a set of cancer profiles, each of length *G*, that represent the tumor profiles purified of normal contamination. Cancer profile ***c***_*n *_corresponds to input tumor profile ***t***_*n*_, where element *g *of the vector ***c***_*n *_(*c_n, g_*) represents the estimate of the relative abundance of transcript *g *in the cancer cell population of tumor *n*. In ISOpure, each vector ***c***_*n *_can be interpreted as a probability distribution over the transcripts; in other words, the elements of ***c***_*n *_are non-negative and sum to one, and *c_n, g _*represents the probability of picking transcript *g *if a random sample is taken from the population of transcripts in the cancerous cell population in tumor *n*.

b) *α*_1_, *α*_2_,..., *α_N _*is a set of 'tumor purity' estimates, where *α_n _*is the estimated fraction of RNA in tumor sample *n *that was contributed by the cancer cells. It can be interpreted as an estimate of the probability that a random transcript from the *n*^th ^(mixed) tumor cell population (represented by ***t***_*n*_) originated from a cancerous cell.

#### Summary of key features of ISOpure

ISOpure employs two main regularization strategies. First, ISOpure assumes that each normal (that is, healthy) profile ***h***_*n *_can be represented by a weighted combination of the available healthy tissue profiles ***b***_1_, ***b***_2_,..., ***b***_*R*_. In other words, ISOpure replaces equation (1) with

(2) $$t_{n}=\alpha_{n}c_{n}+\sum_{r=1}^{R}\theta_{n,r}b_{r}+e_{n},$$

where *θ*_*n*,1_, *θ*_*n*,2_,..., *θ_n, R _*are parameters fit by ISOpure. It further requires that these new parameters are non-negative and that

$$\alpha_{n}+\sum_{r=1}^{R}\theta_{n,r}=1.$$

Thus, *θ*_*n, r *_can be interpreted as the proportion of the transcripts in the *n*^th ^tumor arising from the 'tissue' represented by profile ***b***_*r*_. (Note that to simplify notation, we occasionally indicate *θ*_*n*,1_, *θ*_*n*,2_,..., *θ_n, R _*and *α_n _*using the vector ***θ***_*n *_of length *R+1 *whose *r*^th ^element is *θ_n, r _*for *r < R+1 *and whose *R+1*^st ^element is set to *α_n_*.) This regularization assumption reduces the number of output parameters to *R+G *(*α_n_, θ*_*n*,1_, *θ*_*n*,2_,..., *θ*_*n, R*-1_, ***c***_*n*_). In our experiments, *R *was at most 50. Although the number of estimated parameters is still greater than the number of observations (*G*) and, therefore, there still remains a continuum of solutions to equation (2) that have zero error, the large reduction in parameter number allows us to apply a weaker regularization to ***c***_*n *_and still avoid over-fitting. This strategy also ensures that the contaminating normal profile ***h***_*n*_, implicitly estimated by the algorithm, is similar to the normal tissue types represented by the input profiles ***b***_*r *_to the algorithm. The other regularization strategy used in ISOpure is that it favors solutions in which the values of ***c***_1_, ***c***_2_,..., ***c***_*N *_are clustered together. It encodes this using a scoring function that encourages ***c***_*n *_to be similar to an estimated 'reference cancer profile', ***m***. In other words, ISOpure assumes that the tumor samples in the same collection have similar expression profiles except for some sample-specific deviations that influence prognosis and response to therapy; this assumption is more accurate when the tumors are of the same subtype (for example, adenocarcinomas of the lung [1]). The vector ***m ***is a parameter of the algorithm that is estimated from the tumor profile data, and itself has a regularization applied to it to bias its estimate toward values that are close to the normal profiles. This modeling choice reflects an assumption that in general, profiles of cancerous tissue are similar (but not identical) to those of the tissue of origin of the tumor type. We have previously reported [14] that this assumption improves the accuracy of tumor purity estimation.

#### Additional estimated parameters of ISOpure

Our regularization strategy incorporates the Dirichlet probability density function into our scoring functions. This choice allows us to use the statistical inference method described below to estimate the parameter values. The Dirichlet distribution is a continuous multivariate distribution over discrete probability distributions (that is, vectors of pre-determined size that contain non-negative elements that sum to one). We use the Dirichlet for both ***θ***_*n *_and ***c***_*n *_because they are both discrete probability distributions. The probability density function associated with the Dirichlet has two parameters (termed hyper-parameters because they are the parameters of distributions over model parameters): a mean vector (which determines the mean of the Dirichlet distribution) and a scalar strength parameter that controls how quickly the score decreases from the mode of the Dirichlet distribution. We also estimate the following hyper-parameters from the tumor data:  ν, *k_n _*(for *n *= 1 to *N*), *k*', and ***ω***. These additional parameters are formally defined below in the statistical model provided in equations (3 to 9), but in brief  ν represents both the mean and strength of a Dirichlet distribution over ***θ***_*n*_; *k_n _*represents the strength parameter of the Dirichlet distribution over ***c***_*n *_given ***m***; *k*' represents the strength parameter of the Dirichlet distribution over ***m***; ***ω ***represents the weights on the normal profiles ***b***_*r *_used to make the weighted combination that forms the mean parameter vector for the Dirichlet distribution over ***m***.

### ISOpure algorithm

As mentioned above, the parameter estimates that achieve the best score for a given regularization strategy typically yield non-zero error (represented by ***e***_*n*_) in reconstructing the tumor profile in equation (2). Regularization strategies therefore must also determine the optimal trade-off between decreasing the error ***e***_*n *_in equation (2) and improving the scores of the parameters (that is, ***c***_*n *_and ***θ***_*n *_for all values of *n*) under the Dirichlet distributions encoding our prior assumptions. We formalize the minimization of error ***e***_*n *_as the maximization of the probability of a count vector ***x***_*n *_(derived by discretization of ***t***_*n*_) under a multinomial distribution whose probability vector over transcripts is

$${\hat{x}}_{n}=\alpha_{n}c_{n}+\sum_{r=1}^{R}\theta_{n,r}b_{r}$$

(that is, ${\hat{x}}_{n}$ is a normalized reconstruction of the tumor profile ***x***_*n *_based on the model parameters). The score of a given parameter setting is the product of the score of the parameters under the Dirichlet prior distributions and the probability of the discretized tumor profiles under the multinomial distribution defined by ${\hat{x}}_{n}$. Maximizing this score is equivalent to MAP estimation under the statistical model described below. Note that we estimate the parameters of all of the tumor profiles simultaneously because some of the model parameters (for example, ***m***) depend on all tumor profiles.

#### ISOpure pre-processing and data transformation

In this step, ISOpure applies some simple transforms to the inputs in order to place them in an appropriate form for the model.

The first transform is to discretize the tumor profiles ***t***_*n *_by rounding each element to the nearest non-negative integer to make the count vector ***x***_*n*_. The statistical model underlying ISOpure interprets the elements of the tumor profile as a count of the number of transcripts of that type (gene or transcript isoform) observed in the sample. Ideally, the tumor profiles should be rescaled so that the total number of observations (that is, the sum of the elements) after discretization is approximately the same across all tumor profiles, in order to balance the influence that each tumor profile has on the shared parameters. In the tumor profiles we used in our experiments, the sum of the elements in each of the discretized profiles after robust multi-array average (RMA) normalization was on the order of 10^7^. Profiles may need to be rescaled before discretization if their sum is much less than this, to ensure adequate precision in the discretization.

The second transform is to divide each normal profile ***b***_*r *_by the sum of its elements. After this transformation, each profile ***b***_*r *_sums to one, allowing them to be interpreted as a discrete probability distribution over transcripts.

#### ISOpure statistical model

The full ISOpure model is defined as follows (the probability density and mass functions of the Dirichlet and Multinomial distributions, respectively, are given in Table 1).

**Table 1:** Probability density and mass functions of probability distributions used to define ISOpure.

**Probability density/mass function**^a, b^

$\text{Dirchlet}\left(x|a\right)=\frac{\Gamma \left(\sum_{k=1}^{K}a_{k}\right)}{\prod_{k=1}^{K}\Gamma \left(a_{k}\right)}\overset{K}{\underset{k=1}{\prod }}x_{k}^{a_{k}-1}$
$\text{Multinomial}\left(y|\pi \right)=\frac{\left(\sum_{k=1}^{K}y_{k}\right)!}{\prod_{k=1}^{K}y_{k}!}\overset{K}{\underset{k=1}{\prod }}\pi_{k}^{y_{k}}$

^a^The parameters ***x, a, y***, and ***π ***are all assumed to be vectors of length *K*. Note that the canonical parameter of the multinomial distribution that represents the number of trials is implicit here (that is, the number of trials here is defined as the sum of all elements of ***y***).

^b^Γ(a) is the gamma function of scalar a.

(3) $$B=\left[b_{1}\cdots b_{R}\right]$$

(4) $${\hat{x}}_{n}=\left[B\;\;\;\;\;\;\;\;c_{n}\right]\theta_{n}$$

(5) $$p\left(\theta_{n}|\nu \right)=\text{Dirichlet}\left(\theta_{n}|\nu \right)$$

(6) $$p\left(x_{n}|B,\theta_{n},c_{n}\right)=\text{Multinomial}\left(x_{n}|{\hat{x}}_{n}\right)$$

(7) $$p\left(c_{n}|k_{n},m\right)=\text{Dirichlet}\left(c_{n}|k_{n}m\right)$$

(8) $$p\left(m|k^{\prime },B,\omega \right)=\text{Dirichlet}\left(m|k^{\prime }B\omega \right)$$

where [ ν***w***] indicates the matrix formed by horizontally appending column vectors and/or matrices  ν and ***w ***and ***M*** ν indicates a matrix-vector product of ***M ***and  ν. We estimate the parameters ***θ***_*n *_(including *α_n_*), ***c***_*n*_, ν, ***m***, *k_n_, k*', and ***ω ***using a two-step approach to maximize the complete likelihood function of this model:

(9) $$L=p\left(m|k^{\prime },B,\omega \right)\prod_{n=1}^{N}p\left(c_{n}|k_{n},m\right)\;\cdot \;p\left(\theta_{n}|\nu \right)\;\cdot \;p\left(x_{n}|B,\theta_{n},c_{n}\right)$$

**Figure 1:**
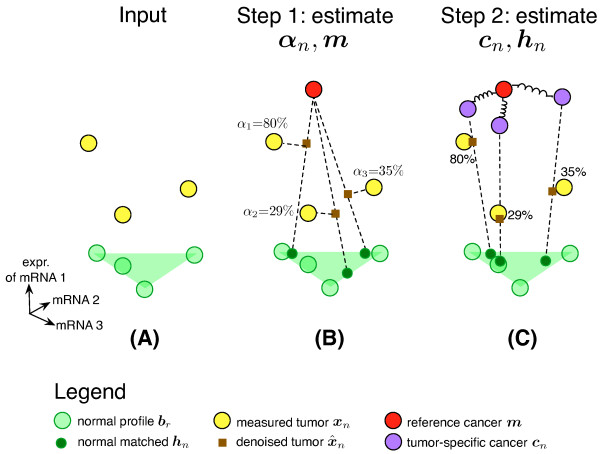
**Schematic of the ISOpure algorithm**. ISOpure is a two-step algorithm for computational purification. **(A) **As input, it takes a set of tumor profiles ***t***_1_, ***t***_2_,..., ***t***_*N *_and a set of normal profiles ***b***_1_, ***b***_2_,..., ***b***_*R*_. It then rounds the component values of each ***t***_*n *_to compute profiles ***x***_*n*_. ISOpure uses the set of normal profiles (green triangle) to estimate the total possible variation in expression due to normal tissue contamination in each tumor sample. **(B) **In Step 1, ISOpure estimates a shared, representative cancer profile ***m***, and the proportion, *α_n_*, of each tumor's mRNA contributed by the cancer cells. **(C) **In Step 2, ISOpure uses ***m ***as the mean of a common Dirichlet prior for each individual cancer profile ***c***_*n *_learned for each input tumor sample ***t***_*n*_, and also estimates ***h***_*n*_, the profile of the contaminating normal tissue in sample *n*.

**Figure 2:**
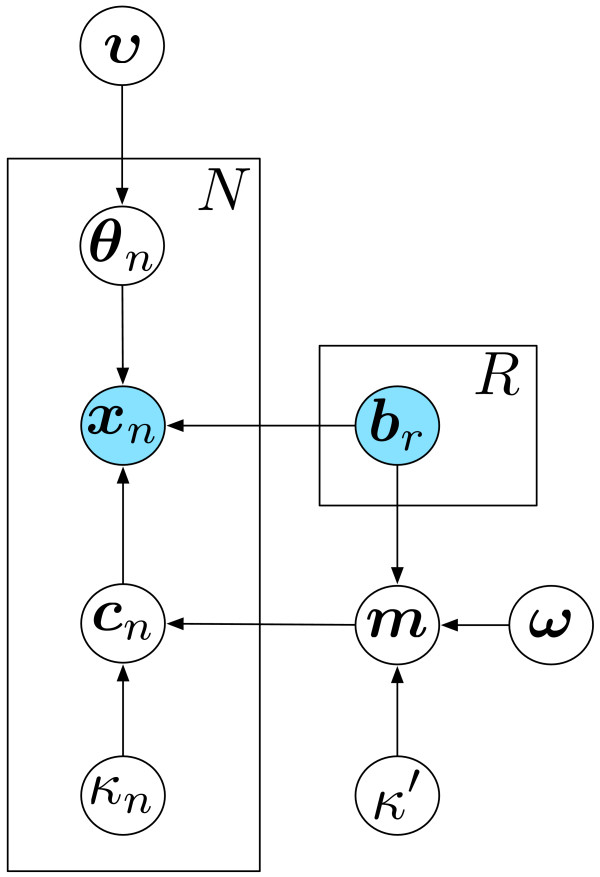
**Graphical model representation of the ISOpure model**. This directed graph represents the joint probability distribution over the input data (blue circles) and the estimated model parameters (white circles), conditioned on the estimated model hyper-parameters (white circles), in the ISOpure model (equations 3 to 9). The conditional probability of each variable depends only on its parents in the graph. To avoid explicitly representing all *N *tumor profiles ***c***_*n *_and *R *normal profiles ***b***_*r *_in the graph (and other associated variables), we used 'plate' notation by drawing one representative node per variable, and enclosing these variables in a plate (rectangular box), where the total number of instances of each enclosed variable is indicated by the fixed constant in the upper right corner of the plate.

Figure 1 shows a geometric interpretation of these two steps. The corresponding probabilistic graphical model illustrating equations (3 to 9) is shown in Figure 2. The first step of ISOpure is similar to previous deconvolution algorithms, and is nearly identical to the ISOLATE (Identification of Sites of Origin by Latent Variables) [14] model we developed previously. The second step of the algorithm is novel. The two regularization strategies of ISOpure greatly reduce the effective number of parameters to be estimated, thereby transforming tumor-specific computational purification into a statistically well-defined estimation problem.

#### ISOpure Step 1: Estimate tumor purities *α*_1_,*α*_2_,...,*α_N _*and the reference cancer profile *m *using the collection of tumor profiles

In this step, ISOpure performs MAP estimation in the statistical model. MAP estimation here is the numerical optimization of a 'complete likelihood' function that is determined based on the underlying statistical model and defined by equation (9). To simplify the optimization and focus on estimating *α*_1_,*α*_2_,...,*α_N _*and ***m***, we initially force all ***c***_*n *_values to be exactly equal to ***m ***(that is, we set *k_n _= ∞ *for all *n *in Step 1). Because all cancer profiles ***c***_*n *_are forced to equal ***m ***throughout Step 1, the estimation of ***m ***tries to simultaneously minimize the reconstruction error ***e***_*n *_(from equation (2)) of all tumors ***t***_*n*_, and therefore the estimates of *α_n _*depend on one another and they are optimized as a group. Note that to perform this estimation we must also estimate *θ*_*n*,1_, *θ*_*n*,2_,..., *θ_n, R_*, although we re-estimate these values in Step 2. We also estimate the hyper-parameters (*k*', ***ω***, and  ν) that specify the Dirichlet distributions over ***m ***and *θ*_*n*,1_, *θ*_*n*,2_,..., *θ_n, R _*and *α_n_*. When doing this, we require that *k*' ≥ 1/*min_r, g _b_r, g _*(where *b_r, g _*indicates the *g*^th ^element of ***b***_*r*_) to ensure that the corresponding Dirichlet density function does not assign infinite density in the limit of one of the elements of ***m ***going towards zero. To estimate our parameters in Step 1 (and Step 2), we run 35 iterations of an optimization procedure that maximizes the complete likelihood function via block coordinate descent from a randomized starting point (see the ISOpure implementation in Additional File 1). Each iteration of this optimization procedure uses the Polak-Ribière conjugate gradient descent method [35] to estimate variables of the same type simultaneously (where we assign the same letter to variables of the same type in equations (3 to 9)) and cycles through each variable type once per iteration. We found that 35 iterations of this optimization procedure yielded a relative change in log likelihood of less than 10^-8 ^between the final two iterations. To find a good local (and possibly global) maximum, we use multiple random initializations (10 in our experiments) and take the one that achieves the highest complete likelihood.

#### ISOpure Step 2: estimate individual cancer profiles *c_n _*for each tumor profile

In this step, we fix *α*_1_, *α*_2_,..., *α_N _*and ***m ***to the values estimated in Step 1, and use MAP estimation to estimate the tumor-specific cancer profiles ***c***_1_, ***c***_2_,..., ***c***_*N *_output by the model, and to re-estimate *θ*_*n*,1_, *θ*_*n*,2_,..., *θ_n, R _*(for all *n*). We also estimate the hyper-parameters *k_n _*and re-estimate  ν; these hyper-parameters specify the Dirichlet distributions over ***c***_*n *_and *θ*_*n*,1_, *θ*_*n*,2_,..., *θ_n, R _*(as described above). Similar to Step 1, we require that *k_n _*≥ 1/*min_g _m_g _*for all *n*. The complete likelihood function for this step is in equation (9), and is optimized using the same algorithm as in Step 1.

#### ISOpure post-processing and data transformation

The main output of the ISOpure implementation in Additional File 1 are the tumor purity estimates *α_n _*and the cancer profiles ***c***_*n*_. To put the estimated profiles on the same scale as the original tumor profiles, we multiply ***c***_*n *_by $S_{n}=\sum_{g=1}^{G}t_{n,g}$.

#### ISOpure summary

The novel contribution of ISOpure over models such as ISOLATE is the ability to estimate per-tumor cancer profiles. ISOpure is freely available as a MATLAB package (Additional File 1) that can also be run in the open-source Octave environment with a small number of modifications; the latest version is maintained here [36]. All ISOpure cancer profiles are available online (see Additional File 2; see Additional File 3; see Additional File 4; see Additional File 5).

### The ISOpure-evenprior algorithm

We hypothesized that the key feature of ISOpure that enables accurate deconvolution is the assumption that the individual cancer profiles ***c***_*n *_are clustered, as encoded by the Dirichlet prior in equation (7). To test this hypothesis, we designed another model, ISOpure-evenprior, that is exactly the same as ISOpure (equations (3 to 9)) except that it replaces the prior defined in equation (7) with one where all the components of ***m ***are replaced with 1/*G *as follows, where **1 **is a column vector of ones of length *G*:

(10) $$p\left(c_{n}|k_{n},m\right)=\text{Dirichlet}\left(c_{n}|k_{n}\frac{1}{G}1\right)$$

### Application of the Clarke method for computational purification

To benchmark the prognostic performance of ISOpure against existing methods, we considered the Clarke [33] and Gosink [34] methods because they are the only existing methods that can be used to estimate per-tumor cancer profiles. Because the Clarke method is designed to be a robust version of the Gosink method, we tested only the Clarke method.

We downloaded the source code for the method from the web site of the authors of this method [37]. We modified the code to implement the knee-finding algorithm as presented in the original paper, as the available code did not implement it, and confirmed using the provided data that the knee-finding algorithm reproduced the same results as the original work. Because none of the tumor datasets processed in this study included matched normal profiles for each tumor, as required by the Clarke method, we used Spearman rank correlation to identify the most similar normal profile ***b***_*r *_for each input tumor sample, and used that normal profile as the matched normal for input into the method. Finally, because the provided code only estimates the tumor purity *α_n _*for each tumor sample *n*, we estimated a tumor-specific cancer profile ***c***_*n *_as suggested by the authors as follows

(11) $$c_{n}=\frac{t_{n}-\left(1-\alpha_{n}\right)b_{f\left(n\right)}}{\alpha_{n}}$$

where *f*(*n*) is the index of the selected matched normal. For our implementation of this algorithm, see Additional File 6.

In our prediction benchmarks, we evaluated the Clarke-based cancer profiles using exactly the same procedure we used to evaluate the ISOpure-estimated cancer profiles, as outlined below in the 'Gene signature identification and testing' section.

### Predicting prognosis using the matrix factorization method

We also tested a matrix factorization-based approach to determine whether mixture proportions estimated by deconvolution algorithms (that cannot estimate individual cancer profiles [14,20,21,26-32]) could still be useful for prognostic prediction. In our prediction benchmarks, we concatenated the mixture proportions estimated by Step 1 of ISOpure for each tumor profile *n *(parameters *θ*_*n*,1_, *θ*_*n*, 2_,..., *θ_n, R_*, and *α_n_*) into a 'mixture proportion profile' vector, then evaluated these mixture proportion profiles for predictive performance in the same manner as we evaluated the ISOpure cancer profiles, as described in the 'Gene signature identification and testing' section below.

### Array data processing

Raw data from the Bhattacharjee study [22] were downloaded in the form of CEL files. These data were pre-processed using the RMA algorithm [38] implemented in the affy package (version 1.22.1) for the R statistical environment (version 2.9.2). Updated ProbeSet mappings to Entrez Gene IDs were used [39] (hgu95av2hsentrezgcdf, version 12.0.0), and only adenocarcinomas of the lung were considered in this study (the predominant histological subtype, and the same subtype represented in all other patient cohorts used here). This dataset included 17 healthy samples, of which 14 were used for purification with ISOpure and three were treated as blind control samples (NL1179, NL1675, and NL1698). Of the 127 lung adenocarcinoma samples, 32 were annotated with tumor cellularity estimates made by two pathologists in the original dataset. For evaluation of ISOpure estimates of tumor purity, we removed 12 samples for which the pathologists' estimates differed by more than one standard deviation (SD) of the differences in their estimates (13.7%), leaving 20 samples for analysis. We did this because the two pathologists differed by as much as 50% in their estimates of cancerous tissue content (see Additional File 7: Figure S1).

Raw data from the Beer [40] study was processed using the same pipeline as the Bhattacharjee study, except that we used ProbeSet mappings to Entrez Gene IDs appropriate to the specific platform used in that study (hu6800hsentrezgcdf version 12.0.0). This dataset comprised 86 tumor samples and 10 healthy samples that were used for purification with ISOpure.

Raw data from each of the four cohorts of the Director's Challenge dataset [41] were separately co-normalized using the RMA algorithm with 49 healthy lung samples profiled on the same platform by Landi and colleagues [42], using the affy package (version 1.24.2) for the R statistical environment (version 2.10.1). Again, updated ProbeSet mappings to Entrez Gene IDs were used (hgu133ahsentrezgcdf version 12.1.0). Associated survival data were downloaded from online supplementary files. From the original set of 443 patients, three patients were removed from the prognostic prediction analysis because of missing data for survival time (NCI_lung216_U133A) or stage (Moff-0683H and Moff-0928E).

Raw data from the Wang study [21] were normalized using the RMA algorithm implemented in the affy package (version 1.26.1) for the R statistical environment (version 2.11.0). To map probe names to Entrez Gene IDs, an updated CDF file was used (hgu133plus2hsentrezgcdf version 13.0.0). After normalization, ISOpure was run on the 109 prostate tumor expression profiles, using 32 biopsy and 13 autopsy samples as the healthy tissue panel (both of which are reported as cancer-free).

Raw data from the Wallace study [43] were normalized using the RMA algorithm implemented in the affy package (version 1.26.1) for the R statistical environment (version 2.11.0). To map probe names to Entrez Gene IDs, an updated CDF file was used (hgu133a2hsentrezgcdf version 13.0.0). This dataset included 69 prostate tumor profiles and 20 healthy profiles, though two healthy profiles were removed because they were collected using pooled RNA samples.

### Gene signature identification and testing

**Figure 3:**
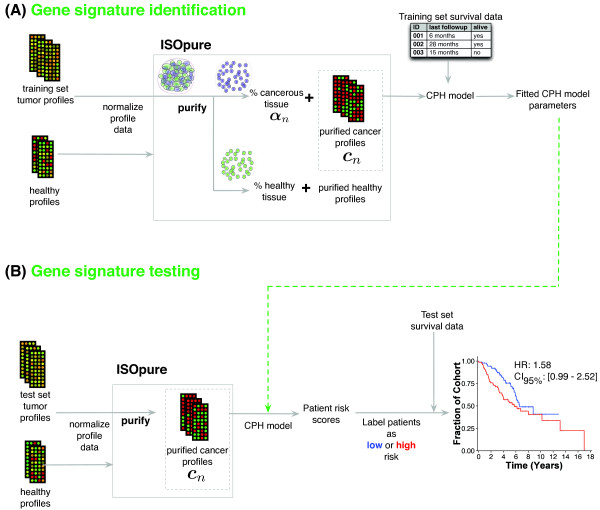
**Pipeline for tumor purification and subsequent identification and testing of gene expression-based prognostic models**. **(A) **Gene signature identification stage. First, profiles from tumor samples and healthy tissue are co-normalized together using the robust multi-array average (RMA) method, then input into ISOpure to estimate the purified cancer profiles ***c***_*n*_. The purified cancer profiles are used as covariates to train an elastic net-regularized Cox proportional hazards (CPH) model (the gene signature) to predict survival data associated with each tumor sample. The trained parameters of the CPH model are used later in model testing. **(B) **Gene signature testing stage. First, new (test-set) tumor profiles are co-normalized with healthy tissue profiles and purified using ISOpure. Each purified cancer profile is then used to compute a risk score for the corresponding patient, using the CPH model parameters learned in the training stage. Patients in a test cohort are then divided into low-risk and high-risk groups based on their risk score, and the hazard ratio is calculated to evaluate the low- and high-risk classifications.

Figure 3 outlines our overall strategy for gene signature identification and testing for the prediction of prognosis of lung cancer patients. In brief, for each benchmark, we trained two elastic net-regularized Cox proportional hazards (CPH) models on the tumor gene expression profiles and the associated survival data. One of these models was trained on unpurified profiles and the other on profiles purified with ISOpure. We use the term 'gene signature' to refer to a learned CPH model that consists of a list of genes and their non-zero regression coefficients. Note that the elastic net regularization applies only to the CPH model, and is separate from the regularization used in the purification procedure. For the lung adenocarcinoma section, we ran two different benchmarks. In the first, we grouped the four independent cohorts of the Director's Challenge dataset into a training dataset of 254 patients (comprised of the HLM and MI cohorts) and testing dataset of 186 patients (comprised of the DFCI and MSKCC cohorts) as previously described [41], using either the ISOpure cancer profiles or the original unpurified profiles. We first median-centered and unit-normalized all gene expression measurements in the training dataset to bring the profiles to the same scale, as previously described [9]. Then, using the glmnet package (version 1.3) [44] on an installation of R (version 2.11.0), we ran five-fold cross-validation using the glmnetCV command with default parameters and *α *= 0.1 to identify the specific gene signature that maximized cross-validation performance on the training dataset. The identified gene signature was re-run on the entire training dataset to find its median risk score, to be used as a threshold for identifying low-risk and high-risk patients in the testing dataset. We then median-centered and unit-normalized all gene expression measurements in the testing dataset, and used the identified gene signature to independently assess risk scores for each of the 186 patients in the testing dataset. These risk scores were used to categorize patients into low-risk or high-risk groups, dichotomized using the median risk score computed on the training dataset. We computed the stage-adjusted hazard ratios and Wald test *P*-values by comparing the survival data of the low-risk versus high-risk patients identified across the testing dataset. Only the gene signature and median risk score were carried over from the analysis of the training dataset, making this testing dataset fully independent.

For the second lung adenocarcinoma benchmark, we used the Beer cohort [40] as a training dataset and all four cohorts from the Director's Challenge as a testing dataset [41], pre-processed and evaluated as described above.

### Prediction of extra-prostatic extension in prostate tumors

As another prediction task, we used prostate tumor expression profiles to predict extra-prostatic extension (EPE) of the prostate tumors, which is a strong predictor for recurrence [45]. Clinically annotated gene expression profiles for 69 prostate tumor and 18 healthy prostate samples were downloaded [43], normalized with RMA, and pre-processed and purified by ISOpure as described in the previous section. EPE is a binary outcome indicating whether or not extension has occurred, therefore, prediction of EPE in prostate tumors is a classification problem. We trained elastic net-regularized logistic regression classifiers using either the original expression profiles, the cancer expression profiles estimated by ISOpure, the estimates of sample composition made by matrix factorization, or the cancer expression profiles estimated by the Clarke method. We used glmnetCV (version 1.3) [44] on R (version 2.11.0) to train each model and to measure the 10-fold cross-validation accuracy. To set the two regularization parameters we conducted a grid search, evaluating each combination of parameters using 10-fold cross-validation on the training set. For each of the 100 λ values selected by glmnet by default, we tried all values of *α *between 0.1 and 0.9, inclusive, with step size of 0.1. We then trained a model using the entire training set with the regularization parameters that led to the highest training accuracy during the grid search. To reduce the effect of fold selection, and allow pairwise comparisons of prediction accuracy between each purification method, each method was trained with identical fold divisions. The entire procedure was repeated 10 times, and the mean accuracy on the held-out folds is reported.

### Measuring prognostic performance as a function of training cohort size

To measure gene signature performance as a function of training cohort size, we repeated the gene signature testing procedure described above using the first lung adenocarcinoma benchmark (that consists of separate training and testing datasets from the Director's Challenge cohorts), except that we sub-sampled (without replacement) tumor samples from the training dataset. For each training cohort size tested, we constructed prognostic models on 1,000 random subsets of the full training dataset, and evaluated model performance on the 186 patients in the test-set cohort. We tested training cohort sizes of 50 to 250 patients, in increments of 10 patients. We used linear interpolation between tested cohort sizes to estimate model performance on other training cohort sizes.

## Results

The ISOpure algorithm is outlined in Figure 1. To evaluate ISOpure, we compared the predictive performance of prognostic models (gene signatures) generated from the original unpurified microarray expression profiles with models generated from the cancer profiles estimated by ISOpure and the Clarke methods, and the mixture proportion profiles from matrix factorization. Figure 3 and Methods outline our procedure for tumor purification, identification of a prognostic gene signature on a training cohort, and testing the gene signature on an independent patient cohort. We selected two tumor types for this evaluation (prostate and non-small cell lung adenocarcinomas) based on the availability of large cohorts [41], and because these tumor types do not yet have established sub-types. We selected prognostic prediction as the clinical task, because in both diseases, treatment escalation/de-escalation is of immediate clinical relevance. The majority of intermediate-risk prostate cancer patients are over-treated, and current therapies such as prostate removal result in serious morbidities. It is predicted that up to a quarter of patients with non-small cell lung cancer would derive benefit from treatment escalation but do not receive it, whereas a similar number of patients classified as stage II are thought to be over-treated [9,46].

### Computational purification improves prognostic gene signatures for lung and prostate cancer

**Figure 4:**
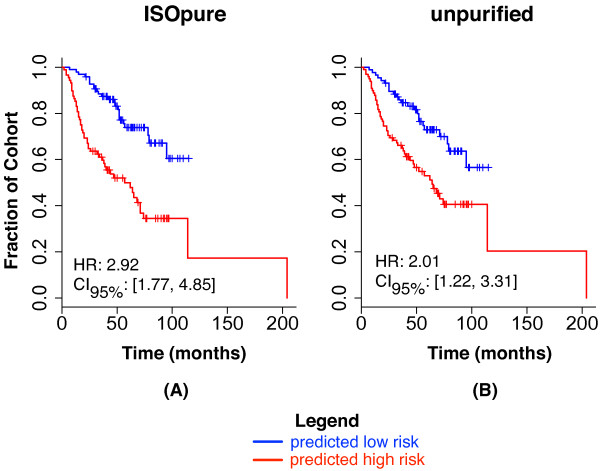
**Test-set performance of CPH models on the MSKCC and DFCI cohorts of the Director's Challenge**. Each plot shows Kaplan-Meier survival curves for the predicted low-risk group (blue) and the predicted high-risk group (red). Plots show the empirical survival probability *S(t) *as a function of time. The hazard ratio (HR) of the high-risk versus the low-risk groups is given, along with its 95% confidence interval. Test-set performance is reported for both the **(A) **ISOpure cancer profiles and **(B) **original, unpurified profiles.

We compared the predictive performance of the unpurified and the ISOpure cancer profiles on the Director's Challenge [41] benchmark of 440 lung adenocarcinomas collected in four cohorts from four different institutions (Figure 4). This benchmark used two of the cohorts (*N *= 254 patients) as a training set, and two other cohorts (*N *= 186 patients) as a held-out, independent test set. We identified separate gene signatures (CPH models) on the unpurified profile and the ISOpure cancer profile training sets, and used them to classify test-set patients into low-risk and high-risk groups. Model performance was measured by the hazard ratio (HR; that is, the relative hazard of death for samples classified in the high-risk group), with a higher value indicating better performance. The ISOpure-based signature (HR = 2.92, *P *= 3.47 × 10^-5^, Wald test) was significantly better at predicting patient outcome (*P *= 0.001, likelihood ratio test) than the unpurified profile-based signature (HR = 2.01, *P *= 0.006, Wald test). Note that the unpurified profile-based signature is an extremely strong baseline for comparison: none of the eight groups in the Director's Challenge generated a gene signature that was significantly better than random. Purifying tumor profiles using representatives of existing expression deconvolution methods (the Clarke method [33] and a matrix factorization-based method [14]) degraded performance compared with the unpurified profiles (HR_clarke _= 1.83, HR_mf _= 1.09) (see Additional File 8: Figure S2).

**Figure 5:**
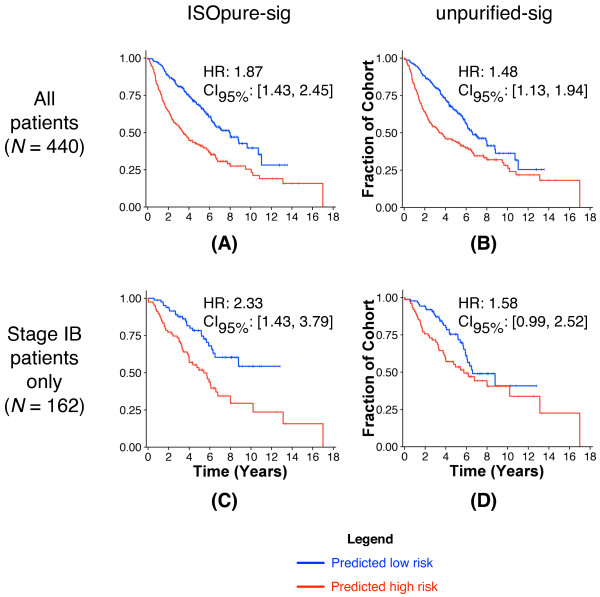
**Test-set performance of CPH models on all four cohorts of the Director's Challenge**. Plots show Kaplan-Meier survival curves as in Figure 4, using CPH models trained on the Beer [40] cohort. Test-set performance of **(A) **ISOpure-sig classification of the ISOpure cancer profiles from the Director's Challenge cohorts and of (**B**) unpurified-sig classification of the unpurified profiles from the Director's Challenge cohorts. **(C) **Same as (A), but the performance is measured only for patients with stage IB cancer. **(D) **Same as (B), but the performance is measured only for patients with stage IB cancer.

To introduce technical variability into the tumor profiles, as may arise in clinical conditions, we repeated our evaluation procedure using a training set [40] collected on a different platform and in a different study from that of the test-set data [41] (Figure 5). This also allowed us to use the full Director's Challenge cohorts (one of the largest lung adenocarcinoma datasets available) as a test set. Again, we found the test-set performance of the ISOpure-based signature of 110 genes (ISOpure-sig; HR = 1.87, *P *= 4.7 × 10^-6^, Wald test) was significantly better (*P *= 2.77 × 10^-4^, likelihood ratio test) than the 82-gene signature based on the unpurified profiles (unpurified-sig; HR = 1.48, *P *= 0.004, Wald test). These two signatures (see Additional File 9: Table S1; see Additional File 10: Table S2) have a significant overlap of 48 genes (*P *= 2.0 × 10^-61^, Fisher's exact test), all of which are in agreement about whether their expression increases or decreases the hazard for death. Note that the larger number of genes in ISOpure-sig is not an indication of bias in favor of that method; the size of each signature is selected automatically by a cross-validation procedure that attempts to maximize training-set performance (see Methods). Expanding the size of the unpurified-sig gene signature to 110 genes decreased its test-set performance (HR = 1.41, *P *= 0.011, Wald test). The improved performance of ISOpure is due to the novel regularization used in the second step of the algorithm: the ISOpure-evenprior model, which replaces the Dirichlet prior for ***c***_*n *_used in the second step of ISOpure with a different, commonly used Dirichlet prior, performs comparably to unpurified-sig (HR = 1.51, *P *= 0.004, Wald test).

Of the 440 test-set samples, 70 were classified differently by ISOpure-sig and unpurified-sig. These 70 samples are significantly enriched for stage IB tumors (*P *= 0.011, Bonferroni-corrected hypergeometric test) (see Additional File 11: Figure S3). When the testing cohort was restricted to stage IB tumors, ISOpure-sig provided an even greater benefit over unpurified profiles (HR_ISOpure-sig _= 2.33, HR_unpurified-sig _= 1.58, *P *= 0.003, likelihood ratio test) (Figure 5). In non-small cell lung adenocarcinomas, the predicted prognosis of early-stage tumors influences the decision of whether to perform adjuvant therapy [46], so these improvements in prognostic prediction for early-stage tumors are relevant to improving patient care. Note also that stage I tumors in this cohort had significantly lower predicted cancer content than those of later stages (see Additional File 12: Figure S4), which may explain the larger difference in performance. We further verified that our ISOpure-sig model was a significant predictor of outcome across all patients with stage I cancer (HR = 1.73, *P *= 0.005, Wald test) (see Additional File 13: Figure S5).

Next, we evaluated ISOpure on prostate tumor data. In prostate cancer, the presence of EPE is a strong predictor for recurrence [45], and also indicates the need for post-operative radiotherapy to maximize survival [47]. Current guidelines for the evaluation of EPE are subjective [48] and can only be applied post-operatively. Accurate, objective assessment of EPE based on biopsies would contribute to optimal patient treatment by prioritizing patients for prostate removal.

Gene expression data from the Wallace study [43] for 69 tumor and 18 normal prostate samples were purified using ISOpure, then used to predict EPE. Because of the small number of samples in the dataset, and the lack of a separate test dataset, we used 10 rounds of 10-fold cross-validation to assess relative performance (Table 2). Classifiers trained on the ISOpure cancer profiles were significantly more accurate than classifiers trained using the original unpurified profiles, the Clarke cancer profiles, or the matrix factorization mixture estimates (all pairwise *P*-values < 0.005, Wilcoxon signed rank test). Additionally, prediction accuracies were not significantly different between the three non-ISOpure methods (all pairwise *P*-values > 0.4).

**Table 2:** Accuracy^a ^of elastic net-regularized models for the prediction of extra-prostatic extension (EPE).

Classifier input	Average accuracy, %
Unpurified expression profiles	61.76 ± 1.64
ISOpure cancer expression profiles	69.12 ± 0.90
Matrix factorization estimates	62.94 ± 0.57
Clarke cancer expression profiles	62.50 ± 1.06

^a^The average accuracy and standard error of the mean over ten 10-fold cross-validation runs are reported for logistic regression classifiers trained using either the original unpurified profiles, ISOpure cancer profiles, matrix factorization mixing proportions, or Clarke cancer profiles.

### ISOpure tumor purity predictions correlate with pathologist estimates

Several tumor datasets provide pathologist estimates of tumor cellularity. We used these estimates as a benchmark for the ISOpure estimate of tumor purity. Note that under accurate purification, we expect tumor purity and tumor cellularity to be correlated but not equal, as tumor purity is an estimate of the proportion of mRNA in the sample contributed by cancerous cells, whereas tumor cellularity is based on area and cell counts. Both cell size and the amount of mRNA per cell can vary considerably between cancer and normal cells [49]. Furthermore, pathologists typically assess a slide of the tumor adjacent or proximal to the sample processed for molecular analysis, whereas ISOpure assesses the purity of the sample processed for molecular analysis [21,22].

**Figure 6:**
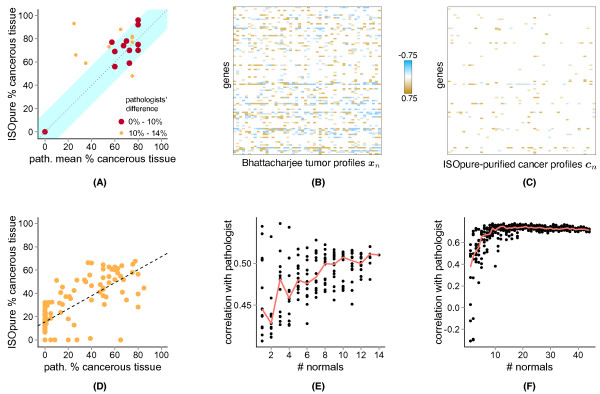
**Comparison of ISOpure-predicted and pathologist reported percentage cancerous tissue**. **(A) **Scatter plot of ISOpure predictions against the average pathologist estimates on a subset of 20 lung tumors and three blinded healthy lung tissues from the Bhattacharjee dataset. The size and color of each point indicate the difference between the pathologists' estimates. The blue region indicates where the ISOpure predictions were within 13.7% of the average pathologist estimate. **(B) **Median-centered expression levels of a random selection of 100 genes in 50 patients of the Bhattacharjee dataset, before ISOpure purification. **(C) **Same genes and patients as in (B), but expression levels were from the ISOpure cancer profiles. **(D) **Scatter plot of ISOpure predictions against a single pathologist on the Wang dataset of 109 prostate tumor samples. The black dashed line indicates the linear regression model that minimizes the sum of squared errors. **(E) **Correlation of ISOpure estimates and the average of the two pathologists' estimates on the same 23 samples as in (A), depicted as a function of the number of normal samples made available to ISOpure. Each point represents a random selection of normal samples of the given size. **(F) **Same as in (E), but correlation was measured for the 109 samples in the Wang dataset.

On a dataset of 20 lung adenocarcinomas and three blinded control samples from Bhattacharjee and colleagues [22] (see Methods), the ISOpure estimates were well correlated (Spearman's ρ = 0.51; *P *= 0.013) with the average of two pathologists (Figure 6A; see Additional File 14: Table S3), although the correlation was lower when the three control samples (that are correctly assigned zero cellularity by ISOpure) were removed (Spearman's ρ = 0.26; *P *= 0.28). Computational purification reduced the inter-tumor variance of all 8,193 gene expression levels in the Bhattacharjee dataset, as expected (Figures 6B, C; see Additional File 15: Figure S6) (*P *< 2.2x10^-16^, Wilcoxon signed rank test).

We also found high correlation between ISOpure and a pathologist on a dataset of 109 prostate samples (Figure 6D, Spearman's ρ = 0.75; *P *= 1.2 × 10^-20^; see Additional File 16: Table S4), although there was a number of samples for which either ISOpure or the pathologist estimated zero cancerous content but the other estimated non-negligible (> 20%) cancerous content. However, predictions made by an independent computational method seemed to be consistent with ISOpure for the tumor samples assigned zero cellularity by ISOpure (see Figure S1B in Wang *et al. *[21]). Furthermore, all of the samples assigned zero cellularity by the pathologist were from surgically removed prostates that were initially diagnosed as cancerous; these assessments of zero cellularity may result from field effects [50] or from the tumor-adjacent slides having negligible cancer content in low-cellularity tumors. As we observed in the lung adenocarcinoma dataset, ISOpure reduced the inter-patient variance in expression for all genes (*P *< 2.2 × 10^-16^, Wilcoxon signed rank test; see Additional File 17: Figure S7).

For both lung and prostate cancer, having a larger set of normal profiles available for purification improved correlation between ISOpure and pathologist cellularity estimates (Figures 6E, F). Correlation for both tumor types saturated at approximately 10 normal profiles. Even with only one normal profile, the ISOpure median correlation was still higher than that of the Clarke method on both the lung (Spearman ρ_ISOpure _= 0.45, ρ_Clarke _= 0.35) and the prostate (Spearman ρ_ISOpure _= 0.38, ρ_Clarke _= 0.19) datasets. Note that matched normal samples were not available for every tumor, as assumed by the Clarke method, so instead we matched each tumor with its most highly correlated normal sample.

## Discussion

Computational purification of tumor expression profiles by ISOpure improves the accuracy of subsequent prognostic models for lung and prostate cancer by reducing inter-sample variation in the amount and type of gene expression signal in the tumor profile that is due to normal tissue contamination. Purifying tumor profiles using other algorithms did not yield classification performance significantly better than the original unpurified profiles (see Additional File 8: Figure S2). Appropriate regularization, embodied by ISOpure priors, was a key factor in this improvement in accuracy; we observed decreased prognostic accuracy with both an unregularized purification method (Clarke) and a modified version of ISOpure with a standard (but inappropriate) prior (see Additional File 18: Figure S8). This modified version of ISOpure used the same tumor purity estimates as ISOpure, and therefore this result indicates that accurate prediction of tumor purity alone is not sufficient for improving prognostic accuracy. Note that although computational purification is related to the well-studied area of expression deconvolution, standard expression deconvolution algorithms do not support profile-specific purification because the only profile-specific information they generate is the tumor purity (more specifically, the proportions of a handful of inferred or provided cell-type specific profiles) [14,20,21,26-32], and these proportions are not strong prognostic indicators in either prostate or lung cancer (Table 2; see Additional File 8: Figure S2). We designed ISOpure as a mixture model that models each tumor sample as a mixture of sample-specific normal and cancer profiles (with some constraints implied by the representative cancer profile and the healthy profiles). Thus, despite substantial efforts in this area, ISOpure is the first validated expression deconvolution algorithm that satisfies a set of reasonable requirements for clinical use of computational purification (Table 3).

**Table 3:** Evaluation of the suitability of ISOpure and other expression deconvolution methods for clinical use.^a.b^

Method	Estimates individual cancer profiles	Uses unmatched normal tissues	Requires minimal additional data	Tested on clinical data
ISOpure	Yes	Yes	Yes	Yes
ISOLATE [14]	No	NA	Yes	No
Erkkila [28]	No	NA	Yes	No
Lahdeskmaki [27]	No	NA	Yes	No
Venet [26]	No	NA	Yes	No
Tolliver [31]	No	NA	Yes	No
Ghosh [29]	No	Yes	No	No
Shen-Orr [30]	No	NA	No	No
Bar-Joseph [32]	No	NA	No	No
Wang [21]	No	NA	No	No
Stuart [20]	No	NA	No	No
Gosink [34]	Yes	No	Yes	No
Clarke [33]	Yes	No	Yes	No

^a^Methods require minimal additional data if they do not require mixing proportions of normal and cancer cells for each input tumor sample for deconvolution.

^b^NA, not applicable; No, negative assessment; Yes, positive assessment.

During preparation of the final revision of our manuscript, we were introduced to the disease-specific genomic analysis (DSGA) algorithm [51], which could, in theory, be adapted to computational purification. DSGA, like ISOpure, models ***h***_*n *_as a linear combination of a set of provided normal profiles but, unlike ISOpure, does not regularize its estimate of ***c***_*n*_. Thus, we suspect that its performance on our benchmarks would be similar to that of ISOpure-evenprior.

Note that the ISOpure regularization strategy is a compromise between previous methods that either over-regularize (and assume all tumor samples are composed of the same small number of cancer and normal cells) or do not regularize (and therefore attempt to solve an ill-posed statistical problem). However, regularization entails making specific assumptions about the nature of the purified cancer expression profiles, and purification quality would probably be reduced if the regularization assumptions were violated.

ISOpure makes two key assumptions. First, it assumes that the set of provided normal profiles contains representative samples of the profiles of the contaminating normal tissue. This assumption could be violated if the set of provided normal profiles does not contain sufficient samples of the type of tissue contaminating the tumors, or if uncorrected batch effects lead to systematic differences in expression between the set of normal profiles and the set of tumor profiles that are not due to cancer. The normal profiles used in the current study were selected to minimize batch effects and we have not evaluated batch-correction procedures with ISOpure. Note, however, that we did not require the profiled normal tissue samples to be from the same patient, and most of the tumor samples we used did not have matching normal profiles available. For prostate and lung, purification using between 10 and 30 normal profiles seemed sufficient; correlation with pathologist estimates of tumor cellularity stopped improving after 10 samples for both tumor types, although the predictive performance of lung cancer prognostic models continued to improve until 30 profiles of normal lung tissue were used (see Additional File 19: Figure S9). The accuracy of the prediction of EPE increased steadily with the number of normal samples available for purification, even when we reached 18 normal samples, the maximum number available (see Additional File 20: Figure S10). These results suggest that collection of more normal samples may have further improved prediction of EPE, although only two normal samples were required to improve EPE prediction significantly above the baseline. In general, our observations suggest that just one or a small number of normal samples will inadequately represent the biological variability in normal gene expression, and collecting as many as 30 normal samples may be necessary to adequately capture normal variation.

ISOpure also assumes that the tumor-specific cancer profiles are similar to the representative cancer profile, ***m***, estimated in the first step. This assumption is appropriate for sets of tumor profiles without strong expression sub-types, such as the prostate and lung datasets we used, but may be violated and lead to decreased performance for cancers with distinct expression sub-types, such as breast cancer. For these cancers, we recommend grouping the tumor profiles by subtype and purifying each group separately. Note that it is possible to extend ISOpure to consider multiple sub-types in the cohort by allowing multiple clusters of cancer profiles. However, doing so would require the estimation of a different '***m*' **vector for each cluster, so in order to avoid over-fitting, the number of inferred sub-types must be much smaller than the number of tumor profiles in the dataset.

The performance of ISOpure is relatively robust against small perturbations in its inputs. With respect to correlation of ISOpure tumor purity estimates with pathologists, the median correlation of ISOpure estimates decreases by 0.01 when using 12 versus 13 normal samples for purifying the lung dataset, whereas the median correlation actually increases by 0.006 when using 43 versus 44 normal samples for purifying the prostate dataset. In terms of prognostic prediction performance, the median accuracy of the ISOpure cancer profiles decreases by 0.015 when using 17 instead of 18 samples for the prostate tumors.

ISOpure-sig is the first validated prognostic signature for the well-studied Director's Challenge benchmark, and it is also prognostic for patients with stage I cancer alone, a group for which good prognostic models are urgently needed for clinical application. The excellent performance of ISOpure for this sub-group may result from the significantly lower cancer content of stage I tumors compared with later stage tumors. This suggests that ISOpure would have similar performance gains on other samples with low cancer content. In addition to improving accuracy for a given patient cohort size, ISOpure can be used to increase the effective size of a patient cohort by reducing inter-patient variability due to contamination. This reduces the cost of cancer biomarker studies and, crucially, enables gene signature identification and application for tumor types for which fewer tumor samples are available (see Additional File 21: Figure S11). We have shown the utility of ISOpure for 834 samples derived from five datasets of two entirely different tumor types. Nevertheless, it is possible that unique features of other diseases or data types will change performance characteristics in different situations, and therefore additional and on-going validation in emerging large datasets [52,53] will be essential.

Our analysis demonstrated approximately 10% improvement in prediction of EPE when using ISOpure cancer profiles compared with the unpurified profiles. EPE prediction is a challenging problem, and we note that without purification, the predictive model performance was only as accurate as simply picking the majority class, so the 10% improvement was actually an increase from zero benefit of considering unpurified expression profiling data. Prostate cancer is the most common malignancy in men, and treatment is often determined entirely by risk groups assigned using pre-treatment prostate-specific antigen levels, biopsy-based Gleason scores, and T category. As a result, improved prediction of EPE from biopsies (that is, improved estimates of T category) could provide benefit when combined with these other risk predictors.

Two requirements must be met in order for ISOpure to be applicable in the clinic. First, collection of gene expression profiles for each patient's tumor sample must become part of the medical diagnosis pipeline. To that end, gene sequence or expression profiling is already being used to inform treatment decisions for multiple cancer types, including breast, gastric, lung, and colorectal cancer [54]. Second, ISOpure relies on expression profiles of normal tissue samples to remove contamination from tumor samples. In the Gene Expression Omnibus (GEO), the number of tumor datasets with associated normal samples is a small subset of the total number of tumor datasets. Collection of a database of normal samples from multiple tissue sites will be needed to use ISOpure; however, as shown by our deconvolution of the Director's Challenge cohort of 443 samples, deconvolution can even use existing datasets of normal samples, if the collection protocol and platform are sufficiently similar.

Although our analysis here focuses on microarray expression profiles, we speculate that ISOpure could be applied directly to abundance estimates from sequencing (RNA-seq) data. Many methods for analyzing RNA-seq transform the reads into a vector of fixed length, whose elements represent abundance estimates, such as RPKM (reads per kilobase per million mapped reads) for genes [55], transcripts (including splice isoforms) [56], or individual exons and exon-exon junctions [57]. These abundance estimates could be directly input into ISOpure as if they are microarray expression profiles, possibly after rescaling them to increase the precision of the discretization of these profiles into count vectors. ISOpure is, however, currently unable to take advantage of data on somatic genetic variants that may help to distinguish reads from normal and tumor RNA. The value of this genetic data may increase with increasing read lengths because the chance that an individual read will cover a polymorphic region will also increase. Future extensions of ISOpure could include these data either as priors on estimates of tumor cellularity or directly as part of the generative model.

Although in this work we focused on addressing inter-tumor heterogeneity due to normal tissue contamination, another source of tumor expression variability is intra-tumor heterogeneity. We expect that when individual tumors contain more than one cancer cell state, the estimated cancer profile of ISOpure would be a weighted average of the different cancer cells contributing to the provided expression profile. However, typical dataset sizes make estimation of even a single cancer profile per tumor sample extremely challenging, and was the focus of ISOpure development. We expect that until multiple samples from each individual tumor can be obtained in a widespread manner, it will not be feasible to address the problem of estimating multiple cancer profiles for each tumor sample because of the small sample sizes. Nonetheless, even with multiple samples per tumor, normal contamination will still be a problem, necessitating the use of tools such as ISOpure. We further suggest that removing the influence of normal contamination may make it easier to distinguish expression patterns unique to sub-clonal populations within the purified profile.

**Figure 7:**
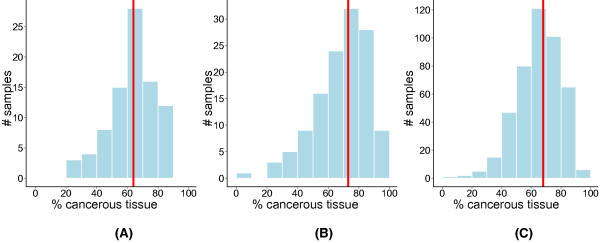
**ISOpure percentage cancerous tissue estimates for 656 lung adenocarcinomas from three datasets**. The median percentage cancerous tissue is shown as a red solid line. **(A) **The Beer dataset (*N *= 86); **(B) **the Bhattacharjee dataset (*N *= 127); (**C**) the four cohorts from the Director's Challenge dataset (*N *= 443).

Sample purification increases the number of patients that can benefit from prognostic models by rescuing samples that otherwise would be discarded because of low cellularity. We and others [20-23,43] have found that tumor samples selected for gene expression analysis vary widely in their cancerous tissue content (Figure 7; see Additional File 14: Table S3; see Additional File 16: Table S4), so a large number of patients stand to gain from improved sample purification. By using ISOpure for purification, prognostic predictions can be made immediately after molecular profiling, at minimal marginal cost. ISOpure can also play an important supplemental role to pathological evaluation of tumors by providing an independent assessment of cancerous RNA content.

## Conclusions

We report a computational purification tool, ISOpure, which mitigates the effects on gene expression profiles of normal tissue contamination in tumor samples and leads to significant improvement in the prediction of patient prognosis and other clinical variables in lung and prostate cancer. The purification, gene signature identification, and testing procedures presented here are fully automated and unbiased, and require only tumor and healthy tissue samples, and associated clinical data (for example, survival or progression indicators). Our procedure can therefore complement any gene signature identification method for any solid tumor, possibly also including those focusing on RNA-seq, protein abundance, or DNA copy-number variation. Although we have chosen here to build upon the architecture of the existing deconvolution algorithm ISOLATE using one particular regularization strategy, other regularization strategies and deconvolution methods may be extended to provide purified, per-tumor cancer expression profiles as well. Although ISOpure has demonstrated success for the analysis of lung adenocarcinoma and prostate tumor samples, future development may be needed to incorporate the possibility of multiple sub-types in a single patient cohort. Nonetheless, we have shown that computational purification methods can improve downstream analyses of tumor expression profiles. We therefore conclude that more exploration of intermediate-strength regularization strategies such as ISOpure may yield significant improvement in downstream analyses of tumor samples and other situations in which biological samples are composed of mixtures of cell types.

## Abbreviations

DSGA: disease-specific genomic analysis; EPE: extra-prostatic extension; HR: hazard ratio; ID: identifier; ISOLATE: Identification of Sites of Origin by Latent Variables; MAP: maximum *a posteriori*; RMA: robust multi-array average; RPKM: reads per kilobase per million mapped reads; SD: standard deviation; CPH: Cox proportional hazards;

## Competing interests

The authors have filed for intellectual property protection on the lung cancer prognostic signature ISOpure-sig.

## Authors' contributions

GQ, PCB, and QM conceived the project and designed the experiments. GQ, AC, and QM designed the ISOpure model. SH, AGD, and PCB collected and pre-processed the gene expression and clinical indicator data. GQ, SH, AGD, and PCB carried out the experiments. All authors analyzed the data and wrote the manuscript. All authors read and approved the final manuscript.

## Supplementary Material

Additional File 1 **ISOpure MATLAB code (ZIP format)**. Click here for file

Additional File 2 **ISOpure cancer profiles from the Beer and Bhattacharjee cohorts, and part of the Shedden cohorts (ZIP format)**. Click here for file

Additional File 3 **ISOpure cancer profiles from part of the Shedden cohorts (ZIP format)**. Click here for file

Additional File 4 **ISOpure cancer profiles from part of the Shedden cohorts (ZIP format)**. Click here for file

Additional File 5 **ISOpure cancer profiles from the Wallace cohort (ZIP format)**. Click here for file

Additional File 6 **Implementation of Clarke's method for estimating tumor purity (R code file)**. Click here for file

Additional File 7 **Figure S1: Comparison of percentage cancerous tissue made by each pathologist on the Bhattacharjee dataset (PDF file)**. The dotted line indicates the *y *= *x *axis, and the blue region indicates where the difference between the estimates of the two pathologists is less than 13.7% (one standard deviation of their overall differences). Click here for file

Additional File 8 **Figure S2: Test-set performance of CPH models on the MSKCC and DFCI cohorts of the Director's Challenge (PDF file)**. We followed the pipeline presented in Figure 3 to train and test gene signatures. We used the Director's Challenge training and testing cohorts as defined in the original study. Illustrated are the test-set performances of CPH models based on **(A) **ISOpure cancer profiles, **(B) **original, unpurified tumor profiles, **(C) **Clarke cancer profiles, and **(D) **matrix factorization mixing proportions (the 50 mixing weights of the cancer and normal profiles estimated by ISOpure Step 1). Performance is adjusted for pathological stage. Click here for file

Additional File 9 **Table S1: Entrez ID and weight of each gene in the 82-gene signature derived from the original, unpurified lung tumor profiles of the Beer cohort (unpurified-sig) (XLS file)**. Click here for file

Additional File 10 **Table S2: Entrez ID and weight of each gene in the 110-gene signature derived from the ISOpure lung cancer profiles of the Beer cohort (ISOpure-sig) (XLS file)**. Click here for file

Additional File 11 **Figure S3: Stage-wise stratification of the patients who were differentially and similarly classified by ISOpure-sig and unpurified-sig (PDF file)**. **(A) **Plot shows the stratification of the 70-patient sub-group classified differently ('diff') by ISOpure-sig and unpurified-sig, and the entire group ('all'). The number of patients in each category is shown above each bar. **(B) **Same as (A), but showing those 370 patients similarly classified between the two signatures ('same'). Click here for file

Additional File 12 **Figure S4: Distributions of percentage cancerous tissue for patients with stage I cancer versus all other stages, computed over all three lung adenocarcinoma datasets (PDF file)**. *N *indicates the number of samples plotted in each box. Six samples were excluded because of missing stage information. Click here for file

Additional File 13 **Figure S5: Test-set performance of CPH models on the 277 stage I patients from the Director's Challenge (PDF file)**. In these prediction experiments, the prognostic models are trained on the Beer cohort, and tested on the stage I patients from the Director's Challenge cohorts. Performance of the prognostic models is based on **(A) **the original unpurified profiles and **(B) **the ISOpure cancer profiles. Click here for file

Additional File 14 **Table S3: Estimates of percentage cancerous tissue made by ISOpure on all three lung adenocarcinoma (Bhattacharjee, Beer, Shedden) datasets (XLS file)**. Click here for file

Additional File 15 **Figure S6: Inter-patient variance of expression levels for 8,193 genes in the Bhattacharjee dataset, before and after ISOpure purification (PDF file)**. The red dashed line is the *y *= *x *line (no change in variance). Click here for file

Additional File 16 **Table S4: Estimates of percentage cancerous tissue made by ISOpure on the Wang prostate dataset (XLS file)**. Click here for file

Additional File 17 **Figure S7: Inter-patient variance of expression levels for 18,185 genes in the Wang dataset, before and after ISOpure purification (PDF file)**. The red dashed line is the *y *= *x *line (no change in variance). Click here for file

Additional File 18 **Figure S8: Test-set performance of a CPH model based on either the unpurified profiles, ISOpure cancer profiles, or ISOpure-evenprior cancer profiles (PDF file)**. ISOpure-evenprior cancer profiles are generated using the same model as ISOpure, except that the Bayesian prior over each individual cancer profile is replaced by a prior whose mean vector is the uniform distribution. **(A) **Test-set performance of a CPH model trained using the Beer cohort and tested on the entire Director's Challenge dataset, when using the original, unpurified tumor profiles. **(B) **Same as (A), but using the ISOpure cancer profiles. (**C**) Same as (A), but using the ISOpure-evenprior cancer profiles. **(D) **Test-set performance of a CPH model trained using the HLM and MI cohorts from the Director's Challenge and tested on the MSKCC and DFCI cohorts from the Director's Challenge, when using the original, unpurified profiles. **(E) **Same as (D), but using the ISOpure cancer profiles. **(F) **Same as (D), but using the ISOpure-evenprior cancer profiles. Performance was adjusted for pathological stage. Click here for file

Additional File 19 **Figure S9: CPH model performance as a function of the number of normal samples for the Director's Challenge dataset (PDF file)**. We followed the pipeline presented in Figure 3 to train and test a gene signature, using the Beer and Director's Challenge datasets as training and testing cohorts, respectively. The full Beer dataset contains 10 normal samples and the full Director's Challenge dataset contains 49 normal samples from the Landi study. The *x*-axis indicates the maximum number of normal samples available to ISOpure for purifying the tumor samples from the training and testing cohorts. Each box shows the distribution of performance for 49 prognostic signatures, each trained with profiles that were purified using a random subset of normal profiles of the indicated size. Because the training cohort only had 10 normal samples, after *x *= 10 we used all 10 normal samples for purification of the training cohort. The *y*-axis indicates the significance of the improvement in performance over the CPH model trained and tested on the unpurified profiles, as measured by the *P*-value from a likelihood ratio test. Click here for file

Additional File 20 **Figure S10: Improvement in extra-prostatic extension (EPE) predictive performance as a function of the number of normal samples (PDF file)**. Predictive power improvement was measured as the difference in accuracy between classifiers trained using the original expression profiles and the ISOpure cancer profiles. For each size of the subset of normal profiles tested, 18 random subsets were drawn from the full set of normal profiles. Click here for file

Additional File 21 **Figure S11: CPH model performance as a function of the training cohort size for the Director's Challenge dataset (PDF file)**. The Director's Challenge cohorts were divided into the same 254-patient training cohort and 186-patient testing cohort used in the original study. Subsets of different sizes of the training cohort were sampled to generate smaller training cohorts, which were then used to identify gene signatures that were evaluated on the full 186-patient testing cohort, as outlined in Figure 3. Results were averaged over 1000 random subsets of each training cohort size and, along with the standard error, are shown for both the ISOpure cancer profiles and the original unpurified profiles. The dotted line indicates the training cohort size required (*N *= 212) for the CPH model based on ISOpure cancer profiles, to achieve the same performance as that achieved by the CPH model based on the original unpurified profiles at a training cohort size of 250 patients. Performance is measured by the hazard ratio (HR), where higher HR is better. Click here for file
