# Supplementary figures and images for: Computational purification of individual tumor gene expression profiles leads to significant improvements in prognostic prediction
Source: Genome Med. 2013 Mar 28;5(3):29. doi: 10.1186/gm433 (PMC3706990; doi:10.1186/gm433)

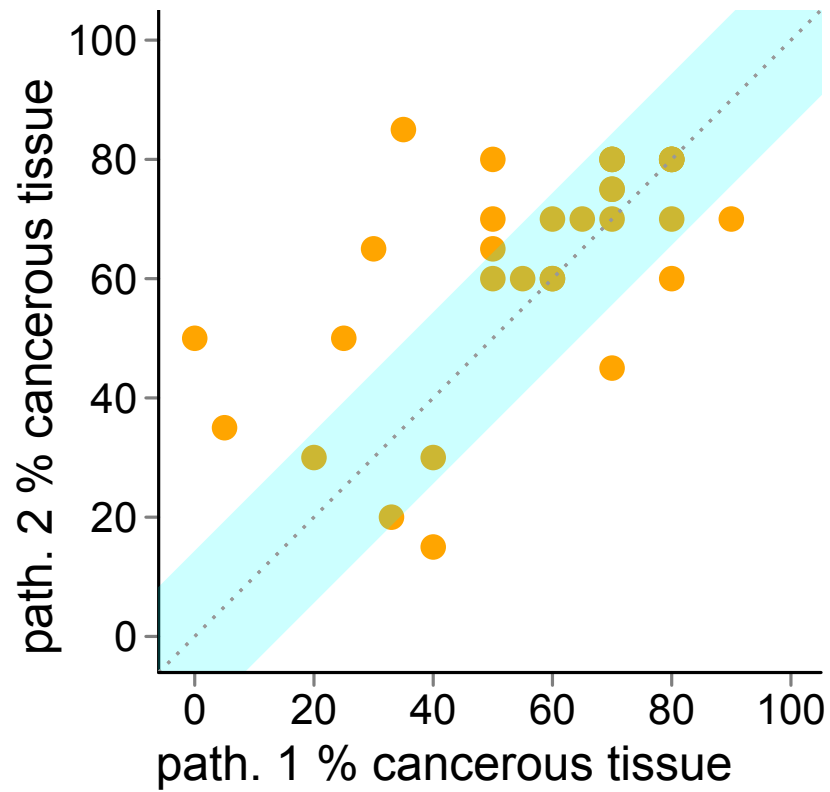

**Additional File 7: Figure S1**

Supplement: Additional File 7 — Figure S1: Comparison of percentage cancerous tissue made by each pathologist on the Bhattacharjee dataset (PDF file). The dotted line indicates the y = x axis, and the blue region indicates where the difference between the estimates of the two pathologists is less than 13.7% (one standard deviation of their overall differences). [file gm433-S7.PDF]

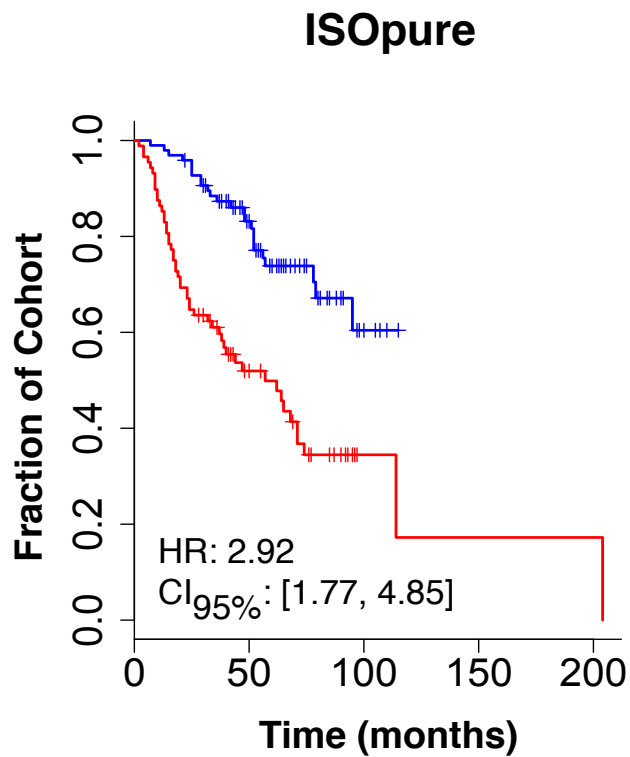

(A)

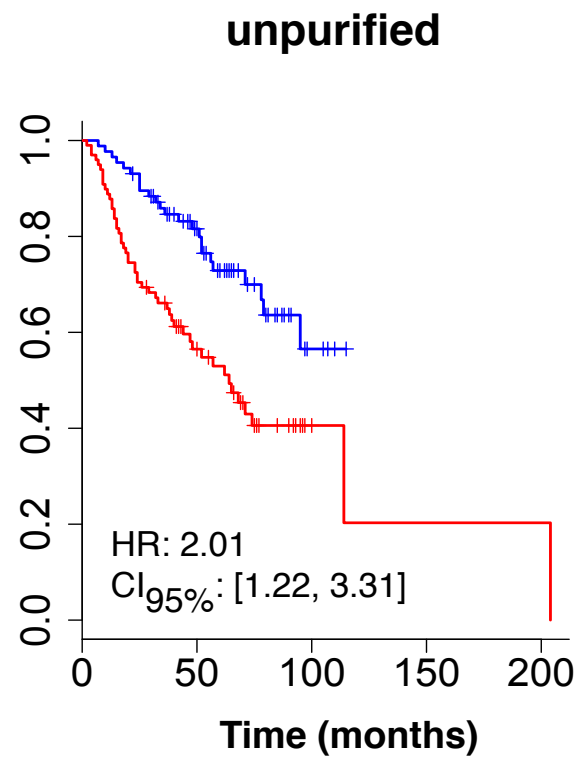

(B)

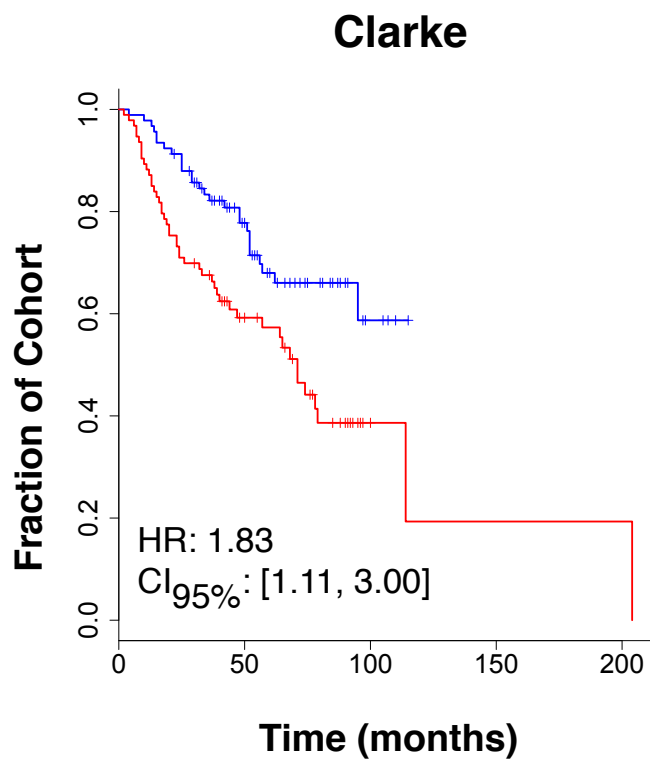

(C)

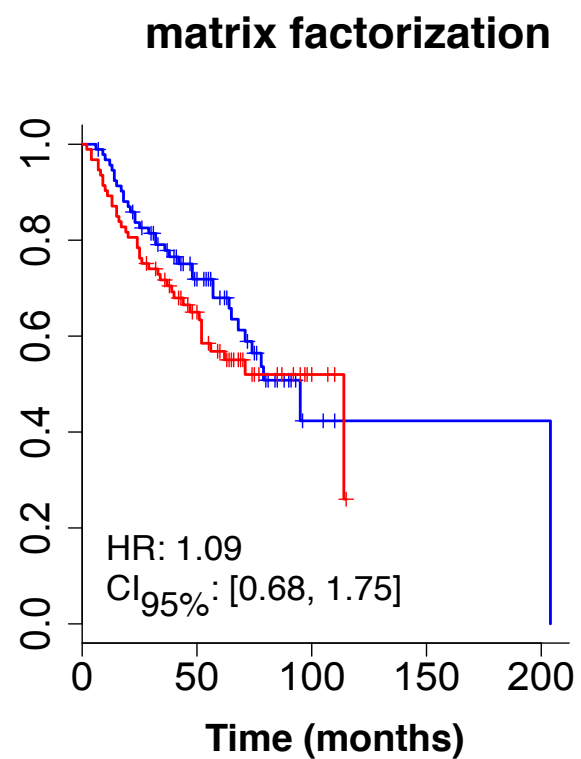

(D)

**Legend**  
— predicted low risk  
— predicted high risk

Supplement: Additional File 8 — Figure S2: Test-set performance of CPH models on the MSKCC and DFCI cohorts of the Director's Challenge (PDF file). We followed the pipeline presented in Figure 3 to train and test gene signatures. We used the Director's Challenge training and testing cohorts as defined in the original study. Illustrated are the test-set performances of CPH models based on (A) ISOpure cancer profiles, (B) original, unpurified tumor profiles, (C) Clarke cancer profiles, and (D) matrix factorization mixing proportions (the 50 mixing weights of the cancer and normal profiles estimated by ISOpure Step 1). Performance is adjusted for pathological stage. [file gm433-S8.PDF]

**(A)**

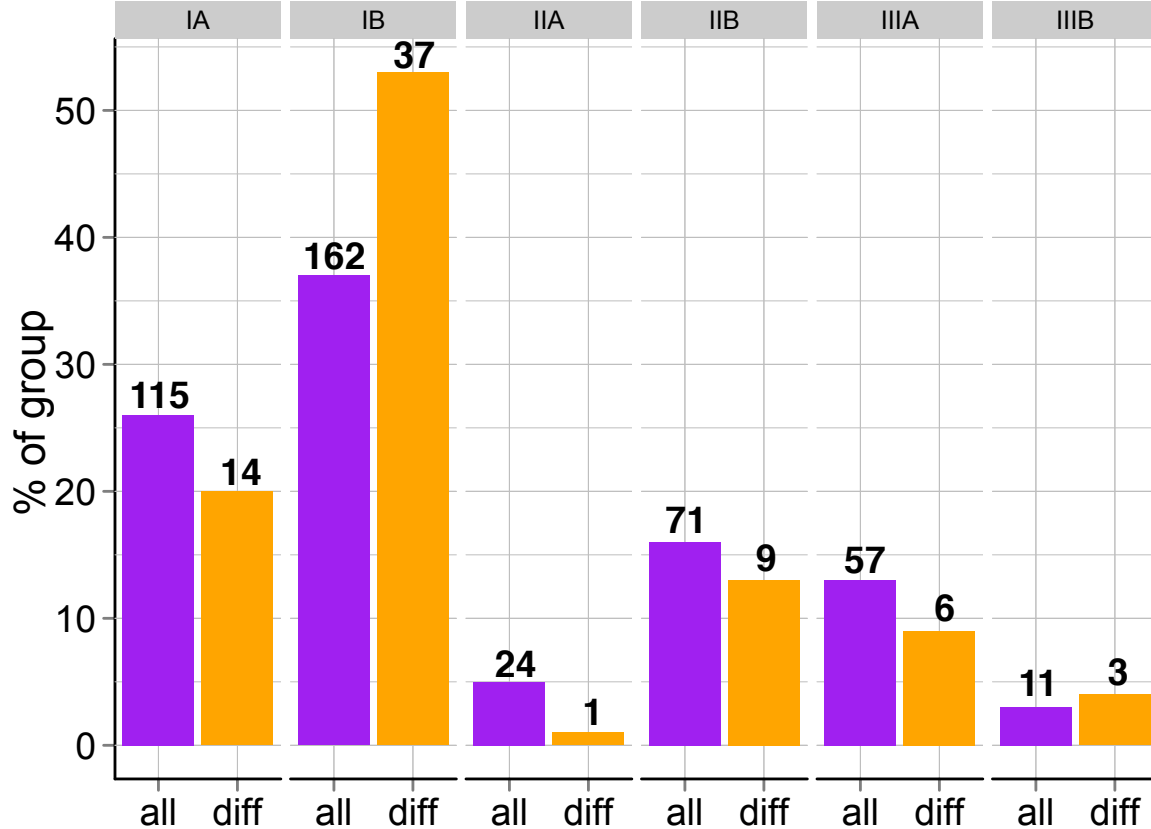

**(B)**

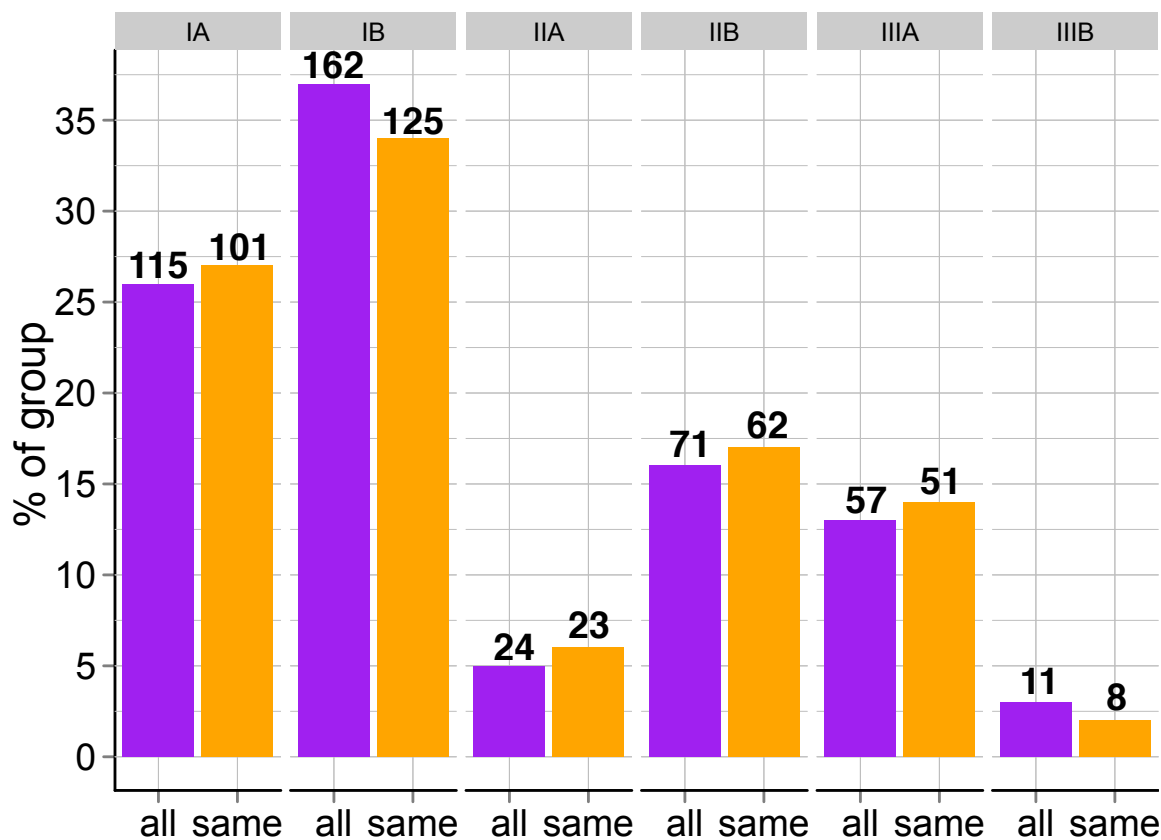

**Additional File 11: Figure S3**

Supplement: Additional File 11 — Figure S3: Stage-wise stratification of the patients who were differentially and similarly classified by ISOpure-sig and unpurified-sig (PDF file). (A) Plot shows the stratification of the 70-patient sub-group classified differently ('diff') by ISOpure-sig and unpurified-sig, and the entire group ('all'). The number of patients in each category is shown above each bar. (B) Same as (A), but showing those 370 patients similarly classified between the two signatures ('same'). [file gm433-S11.PDF]

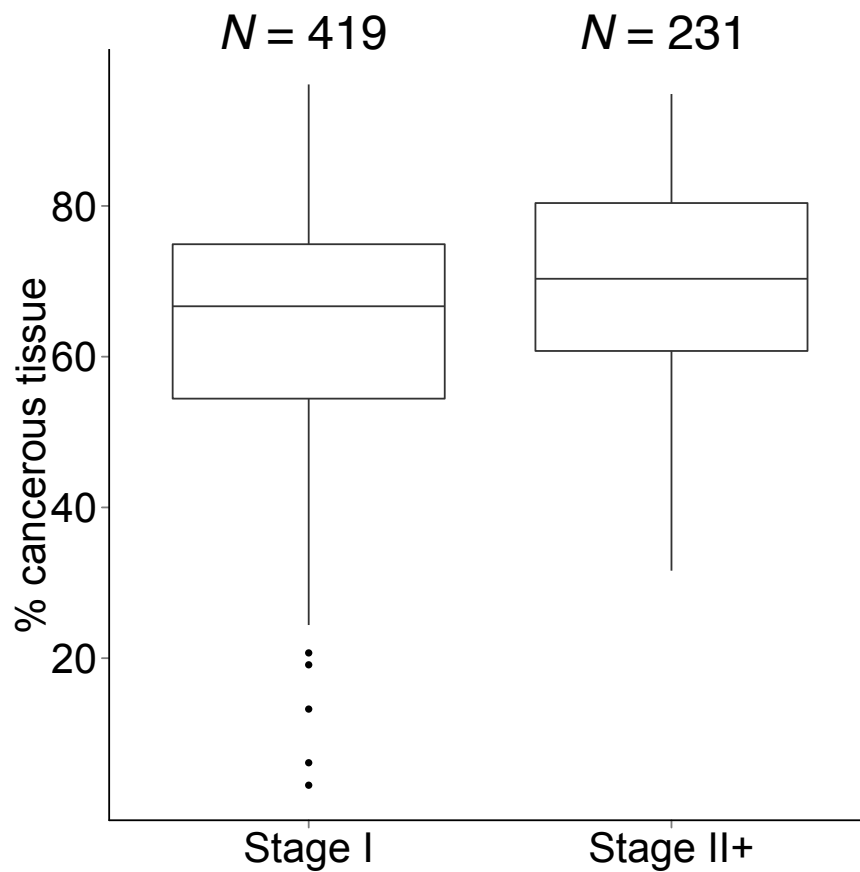

**Additional File 12: Figure S4**

Supplement: Additional File 12 — Figure S4: Distributions of percentage cancerous tissue for patients with stage I cancer versus all other stages, computed over all three lung adenocarcinoma datasets (PDF file). N indicates the number of samples plotted in each box. Six samples were excluded because of missing stage information. [file gm433-S12.PDF]

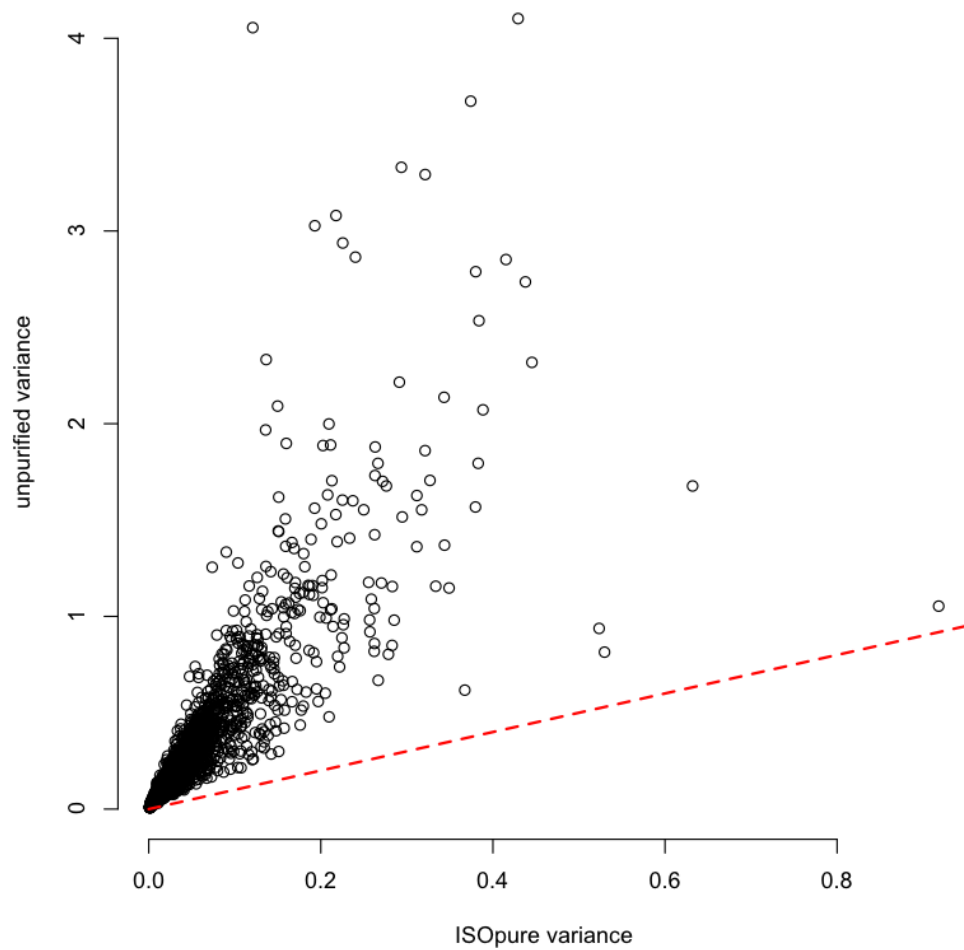

**Additional File 15: Figure S6**

Supplement: Additional File 15 — Figure S6: Inter-patient variance of expression levels for 8,193 genes in the Bhattacharjee dataset, before and after ISOpure purification (PDF file). The red dashed line is the y = x line (no change in variance). [file gm433-S15.PDF]

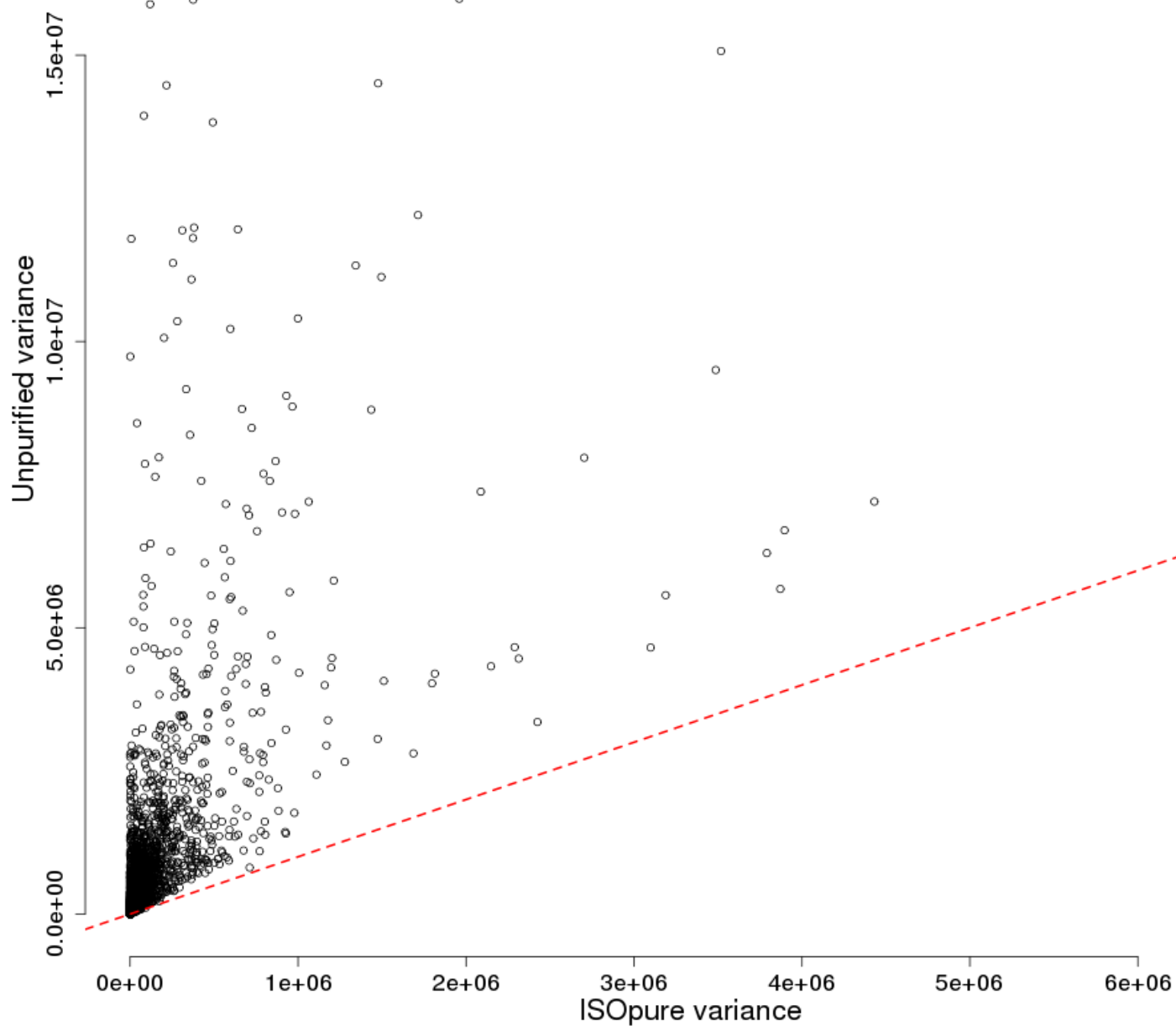

**Additional File 17: Figure S7**

Supplement: Additional File 17 — Figure S7: Inter-patient variance of expression levels for 18,185 genes in the Wang dataset, before and after ISOpure purification (PDF file). The red dashed line is the y = x line (no change in variance). [file gm433-S17.PDF]

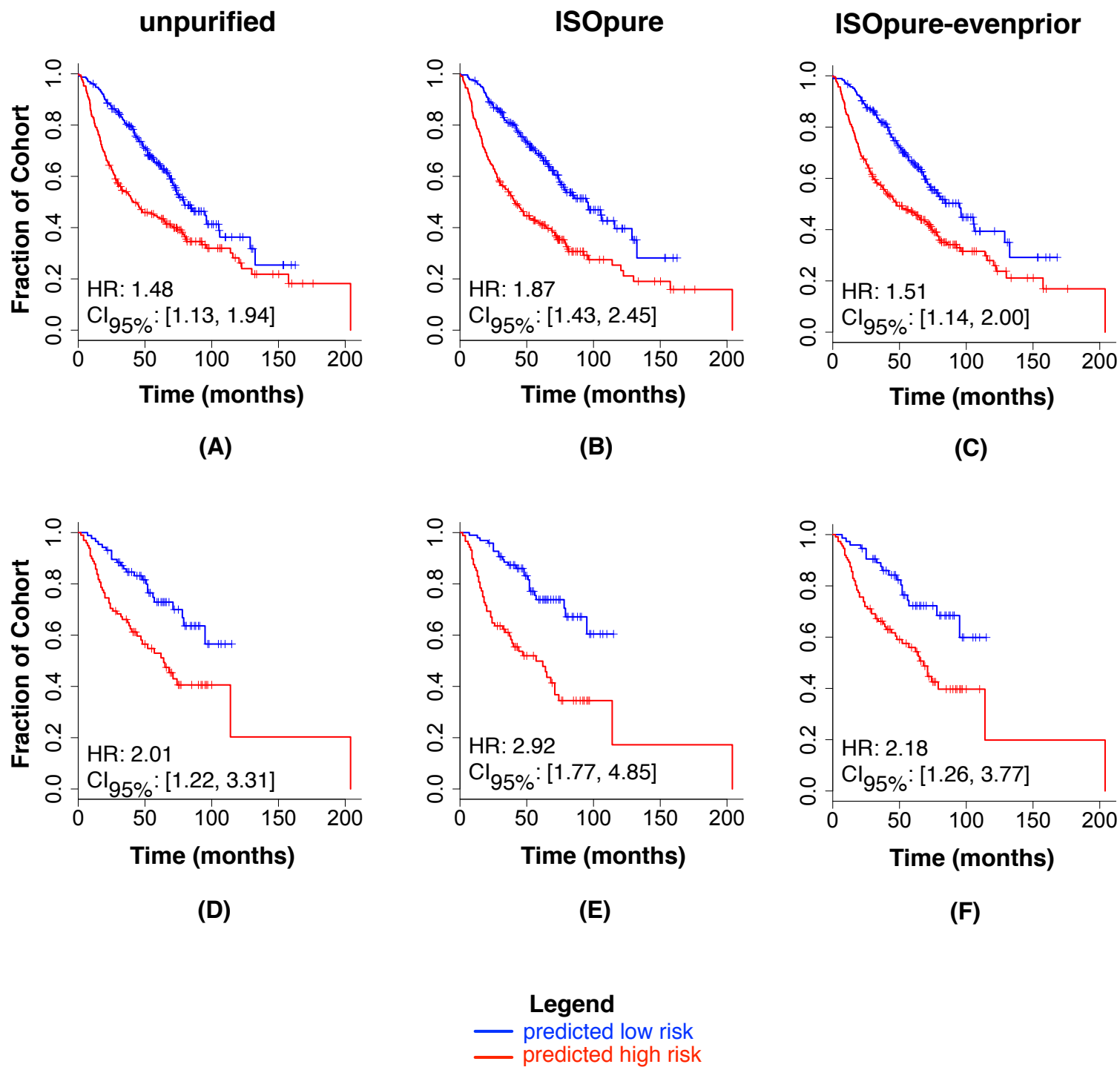

**Additional File 18: Figure S8**

Supplement: Additional File 18 — Figure S8: Test-set performance of a CPH model based on either the unpurified profiles, ISOpure cancer profiles, or ISOpure-evenprior cancer profiles (PDF file). ISOpure-evenprior cancer profiles are generated using the same model as ISOpure, except that the Bayesian prior over each individual cancer profile is replaced by a prior whose mean vector is the uniform distribution. (A) Test-set performance of a CPH model trained using the Beer cohort and tested on the entire Director's Challenge dataset, when using the original, unpurified tumor profiles. (B) Same as (A), but using the ISOpure cancer profiles. (C) Same as (A), but using the ISOpure-evenprior cancer profiles. (D) Test-set performance of a CPH model trained using the HLM and MI cohorts from the Director's Challenge and tested on the MSKCC and DFCI cohorts from the Director's Challenge, when using the original, unpurified profiles. (E) Same as (D), but using the ISOpure cancer profiles. (F) Same as (D), but using the ISOpure-evenprior cancer profiles. Performance was adjusted for pathological stage. [file gm433-S18.PDF]

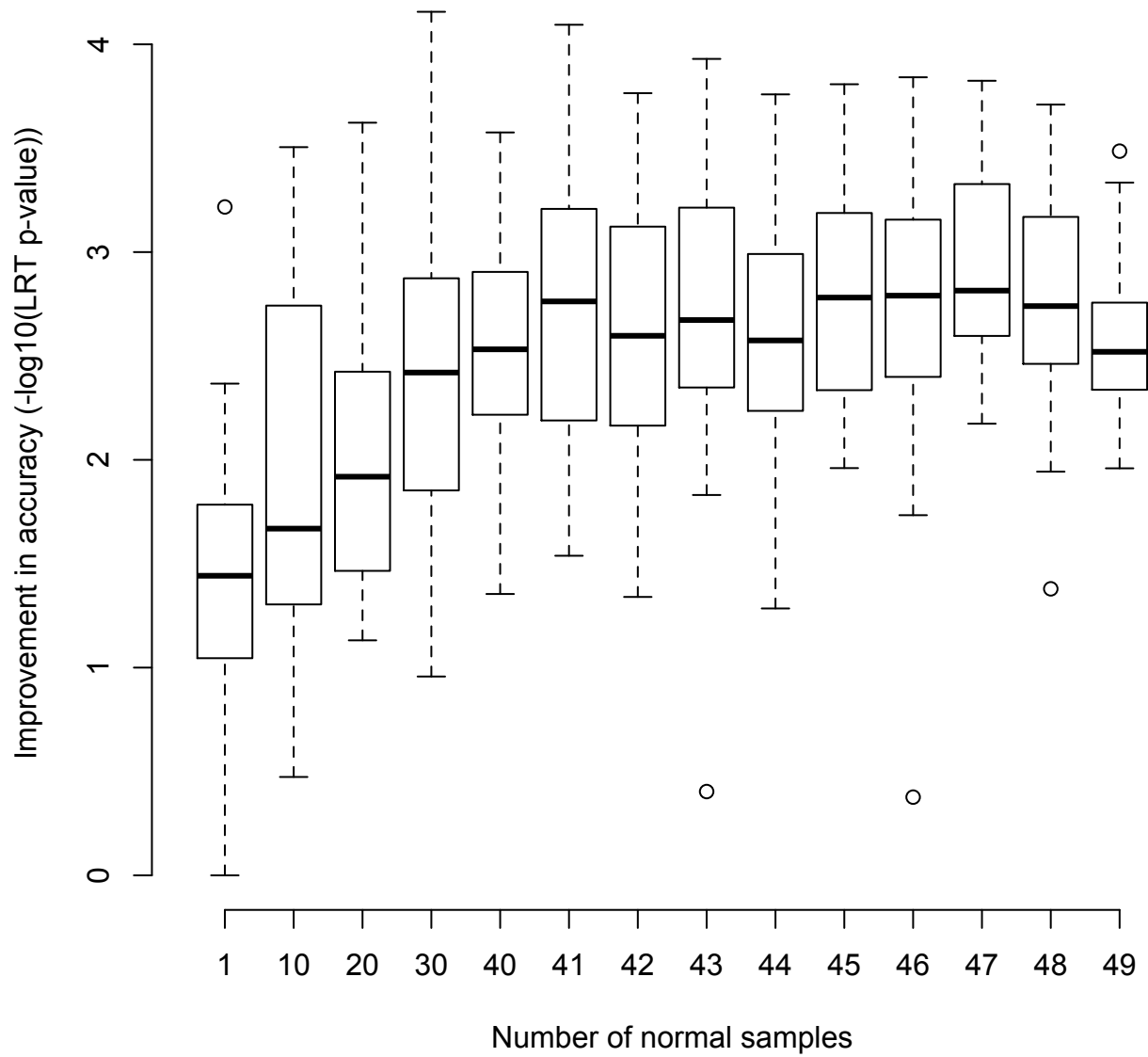

**Additional File 19: Figure S9**

Supplement: Additional File 19 — Figure S9: CPH model performance as a function of the number of normal samples for the Director's Challenge dataset (PDF file). We followed the pipeline presented in Figure 3 to train and test a gene signature, using the Beer and Director's Challenge datasets as training and testing cohorts, respectively. The full Beer dataset contains 10 normal samples and the full Director's Challenge dataset contains 49 normal samples from the Landi study. The x-axis indicates the maximum number of normal samples available to ISOpure for purifying the tumor samples from the training and testing cohorts. Each box shows the distribution of performance for 49 prognostic signatures, each trained with profiles that were purified using a random subset of normal profiles of the indicated size. Because the training cohort only had 10 normal samples, after x = 10 we used all 10 normal samples for purification of the training cohort. The y-axis indicates the significance of the improvement in performance over the CPH model trained and tested on the unpurified profiles, as measured by the P-value from a likelihood ratio test. [file gm433-S19.PDF]

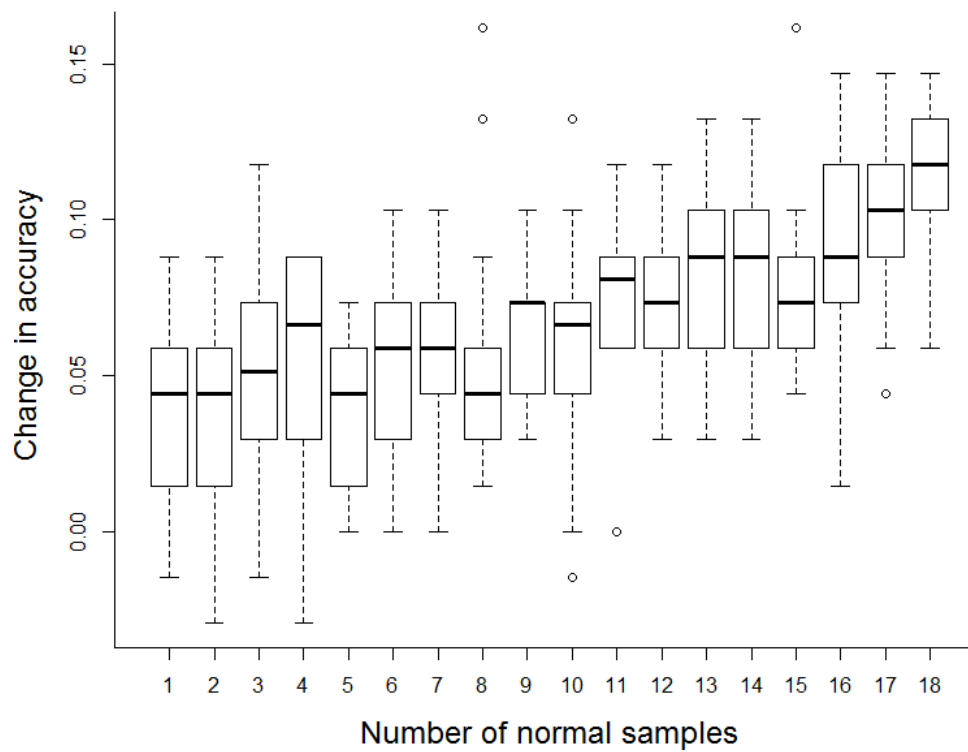

**Additional File 20: Figure S10**

Supplement: Additional File 20 — Figure S10: Improvement in extra-prostatic extension (EPE) predictive performance as a function of the number of normal samples (PDF file). Predictive power improvement was measured as the difference in accuracy between classifiers trained using the original expression profiles and the ISOpure cancer profiles. For each size of the subset of normal profiles tested, 18 random subsets were drawn from the full set of normal profiles. [file gm433-S20.PDF]

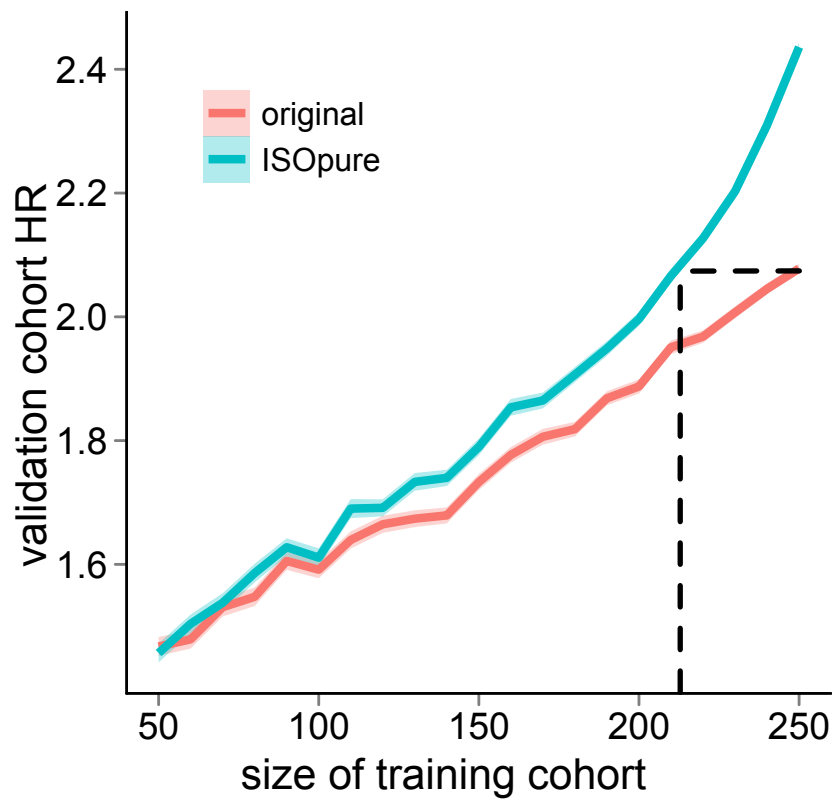

**Additional File 21: Figure S11**

Supplement: Additional File 21 — Figure S11: CPH model performance as a function of the training cohort size for the Director's Challenge dataset (PDF file). The Director's Challenge cohorts were divided into the same 254-patient training cohort and 186-patient testing cohort used in the original study. Subsets of different sizes of the training cohort were sampled to generate smaller training cohorts, which were then used to identify gene signatures that were evaluated on the full 186-patient testing cohort, as outlined in Figure 3. Results were averaged over 1000 random subsets of each training cohort size and, along with the standard error, are shown for both the ISOpure cancer profiles and the original unpurified profiles. The dotted line indicates the training cohort size required (N = 212) for the CPH model based on ISOpure cancer profiles, to achieve the same performance as that achieved by the CPH model based on the original unpurified profiles at a training cohort size of 250 patients. Performance is measured by the hazard ratio (HR), where higher HR is better. [file gm433-S21.PDF]
