# Supplementary material for: Computational purification of individual tumor gene expression profiles leads to significant improvements in prognostic prediction
Source: Genome Med. 2013 Mar 28;5(3):29. doi: 10.1186/gm433 (PMC3706990; doi:10.1186/gm433)
Supplement: Additional File 13 — Figure S5: Test-set performance of CPH models on the 277 stage I patients from the Director's Challenge (PDF file). In these prediction experiments, the prognostic models are trained on the Beer cohort, and tested on the stage I patients from the Director's Challenge cohorts. Performance of the prognostic models is based on (A) the original unpurified profiles and (B) the ISOpure cancer profiles. [file gm433-S13.PDF]

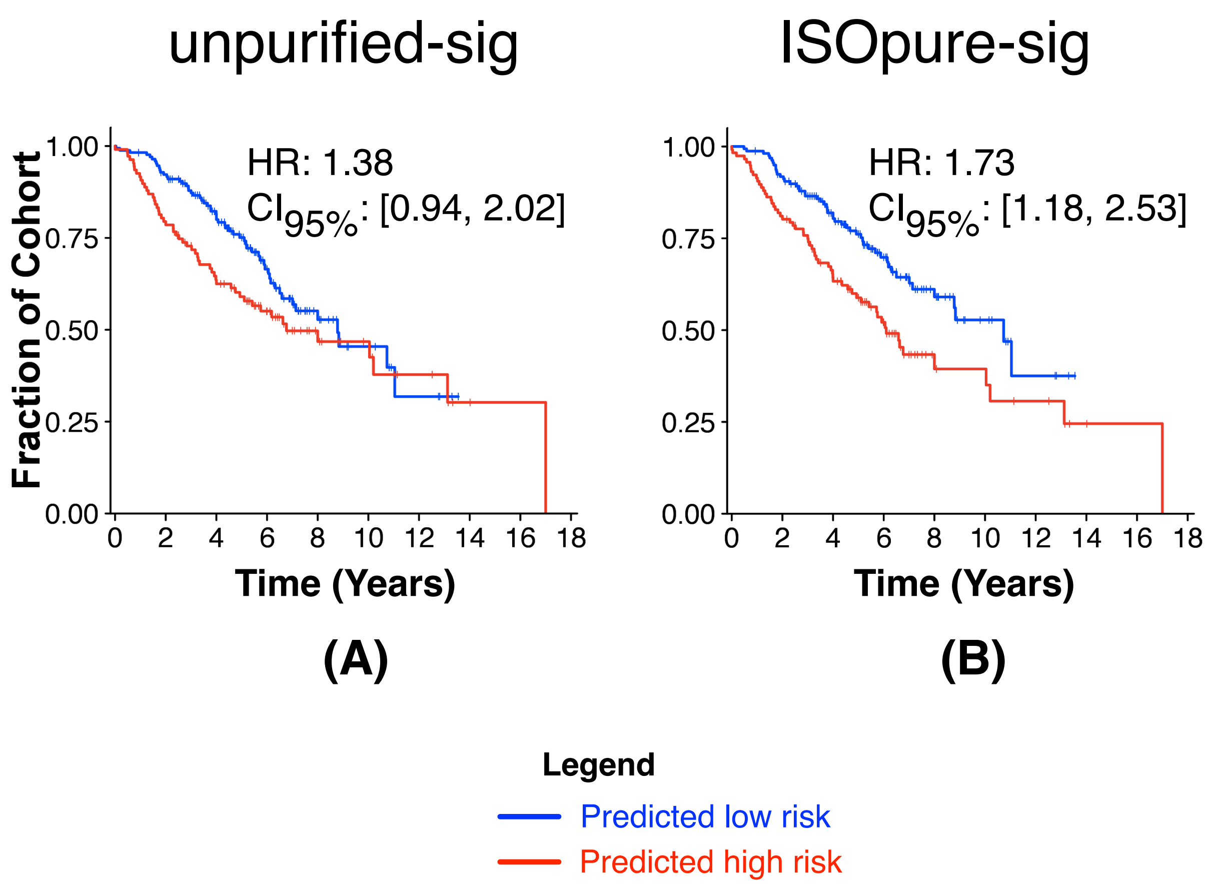

Additional File 13: Figure S5
